# TFEB drives mTORC1 hyperactivation and kidney disease in Tuberous Sclerosis Complex

**DOI:** 10.1038/s41467-023-44229-4

**Published:** 2024-01-09

**Authors:** Nicola Alesi, Damir Khabibullin, Dean M. Rosenthal, Elie W. Akl, Pieter M. Cory, Michel Alchoueiry, Samer Salem, Melissa Daou, William F. Gibbons, Jennifer A. Chen, Long Zhang, Harilaos Filippakis, Laura Graciotti, Caterina Miceli, Jlenia Monfregola, Claudia Vilardo, Manrico Morroni, Chiara Di Malta, Gennaro Napolitano, Andrea Ballabio, Elizabeth P. Henske

**Affiliations:** 1 Pulmonary and Critical Care Medicine, Department of Medicine, Brigham and Women’s Hospital, Harvard Medical School, Boston, MA USA; 2 Section of Experimental and Technical Sciences, Department of Biomedical Sciences and Public Health, School of Medicine, Università Politecnica delle Marche, Ancona, Italy; 3 Telethon Institute of Genetics and Medicine, Naples, Italy; 4 Section of Neuroscience and Cell Biology, Department of Experimental and Clinical Medicine, School of Medicine, Università Politecnica delle Marche, Ancona, Italy; 5 Medical Genetics Unit, Department of Medical and Translational Science, Federico II University, Naples, Italy; 6 SSM School for Advanced Studies, Federico II University, Naples, Italy; 7 Department of Molecular and Human Genetics, Baylor College of Medicine, Houston, TX USA; 8 Jan and Dan Duncan Neurological Research Institute, Texas Children’s Hospital, Houston, TX USA

**Keywords:** Cancer genetics, Paediatric cancer, Cell biology

## Abstract

Tuberous Sclerosis Complex (TSC) is caused by TSC1 or TSC2 mutations, leading to hyperactivation of mechanistic target of rapamycin complex 1 (mTORC1) and lesions  in multiple organs including lung (lymphangioleiomyomatosis) and kidney (angiomyolipoma and renal cell carcinoma). Previously, we found that TFEB is constitutively active in TSC. Here, we generated two mouse models of TSC in which kidney pathology is the primary phenotype. Knockout of TFEB rescues kidney pathology and overall survival, indicating that TFEB is the primary driver of renal disease in TSC. Importantly, increased mTORC1 activity in the TSC2 knockout kidneys is normalized by TFEB knockout. In TSC2-deficient cells, Rheb knockdown or Rapamycin treatment paradoxically increases TFEB phosphorylation at the mTORC1-sites and relocalizes TFEB from nucleus to cytoplasm. In mice, Rapamycin treatment normalizes lysosomal gene expression, similar to TFEB knockout, suggesting that Rapamycin’s benefit in TSC is TFEB-dependent. These results change the view of the mechanisms of mTORC1 hyperactivation in TSC and may lead to therapeutic avenues.

## Abstract (web summary)

Tuberous Sclerosis Complex (TSC) is caused by TSC1 or TSC2 mutations, leading to hyperactivation of mechanistic target of rapamycin complex 1 (mTORC1) and tumors in multiple organs. Here, the authors show that TFEB is the primary driver of renal disease and mTORC1 hyperactivation in TSC.

## Introduction

Tuberous Sclerosis Complex (TSC) is an autosomal dominant disease caused by germline loss-of-function mutations in the *TSC1* or *TSC2* genes, leading to neurologic disease (seizures, autism, and cognitive disability) and lesions in multiple organs including the brain, skin, heart, lungs (lymphangioleiomyomatosis), and kidneys^1,2^. Renal disease, which includes angiomyolipoma, cysts, and renal cell carcinoma, is the major cause of TSC-associated death^3^.

The TSC protein complex (TSC1, TSC2, TBC1D7) integrates growth signals to regulate the activity of mechanistic/mammalian target of rapamycin complex 1 (mTORC1), a key metabolic hub^4–7^. The activation of mTORC1 requires two different types of small GTPases: the Rag GTPases, a heterodimeric protein complex composed of RagA or RagB bound to RagC or RagD, which mediate the nutrient-dependent recruitment of mTORC1 to the lysosomal surface^8–10^; and Rheb, which is activated by growth factors and acts as an allosteric activator of mTORC1^11–13^. Upon growth factor removal, the TSC complex stimulates the conversion of Rheb to its inactive GDP-bound state, thereby inhibiting mTORC1^11,14^. In *TSC1*- or *TSC2*-deficient cells, Rheb is constitutively GTP-bound and mTORC1 is hyperactive^11,14^. This Rag-Rheb-dependent mechanism of mTORC1 activation is required for the phosphorylation of the well-characterized substrates S6K and 4E-BP1. Unlike S6K and 4EBP1, which contain a TOS motif (TOR signaling motif) and are recruited by mTORC1 via its regulatory subunit Raptor^15,16^, TFEB and TFE3, which are master transcriptional modulators of lysosomal biogenesis and autophagy^17,18^, lack a TOS motif and are recruited by mTORC1 via a non-canonical mechanism that requires active RagC/D GTPases but not Rheb^19–21^.

It is well-established that mTORC1 phosphorylates TFEB at serine 211 and TFE3 at serine 321, leading to 14-3-3 protein binding and cytoplasmic sequestration^19,22–24^. Surprisingly, this mechanism does not apply to *TSC1/2* deficient cells: we and others have demonstrated that in TSC, in which mTORC1 hyperactivity is the widely acknowledged hallmark, TFEB, and TFE3 are unexpectedly hypophosphorylated at their mTORC1 sites, leading to nuclear localization and hyperactivation^25–28^. Constitutive activation of TFEB and TFE3 promote cell proliferation in several types of cancer^29^. Chromosomal translocations involving TFEB and TFE3 lead to TFEB/TFE3 overactivation causing renal cell carcinoma^30^. Furthermore, kidney-specific genetic inactivation of TFEB rescues renal disease in a mouse model of Birt-Hogg-Dubé (BHD) syndrome^21^, which is caused by loss-of-function mutations of the RagC/D GTP-ase activating protein folliculin (FLCN) and is characterized by skin tumors, lung cysts, and kidney cancer^31^. Given the prominent role of TFEB in tumorigenesis in other diseases^32^, we sought to determine whether TFEB contributes to the phenotypes associated with TSC.

We report here that TFEB knockout rescues renal pathology and survival in two models of whole-body and kidney epithelium-specific inactivation of *Tsc2*. TFEB inactivation was sufficient to normalize the phosphorylation of mTOR substrates 4E-BP1 and p70 S6 Kinase (S6K) as well as the expression of Rag-Ragulator complex components, critical for the recruitment of mTOR to the lysosomal surface. Moreover, TFEB knockout was as efficient as Rapamycin treatment in decreasing kidney cystogenesis and kidneys to body weight ratio in *Tsc2*-deficient mice. In vitro, we found that Rapamycin treatment paradoxically increased phosphorylation of TFEB at Serine 211 and promoted its cytoplasmic localization in *TSC*-deficient cells in a Rag-dependent manner. In vivo, Rapamycin treatment has a similar RNA-Seq signature compared to TFEB knockout since of the genes upregulated in *TSC2*-deficient kidneys, more than 50% are both TFEB and Rapamycin-dependent, suggesting that Rapamycin effect in TSC is at least partially due to its inhibition of TFEB. Collectively, our data support a concept of “TFE-opathies” in which TFEB and/or TFE3 drive kidney pathology in three distinct diseases: TSC, BHD and translocation RCC^21,25,31,33,34^.

## Results

### TFEB knockout rescues renal pathology in a whole-body inducible model of TSC

**Fig. 1:**
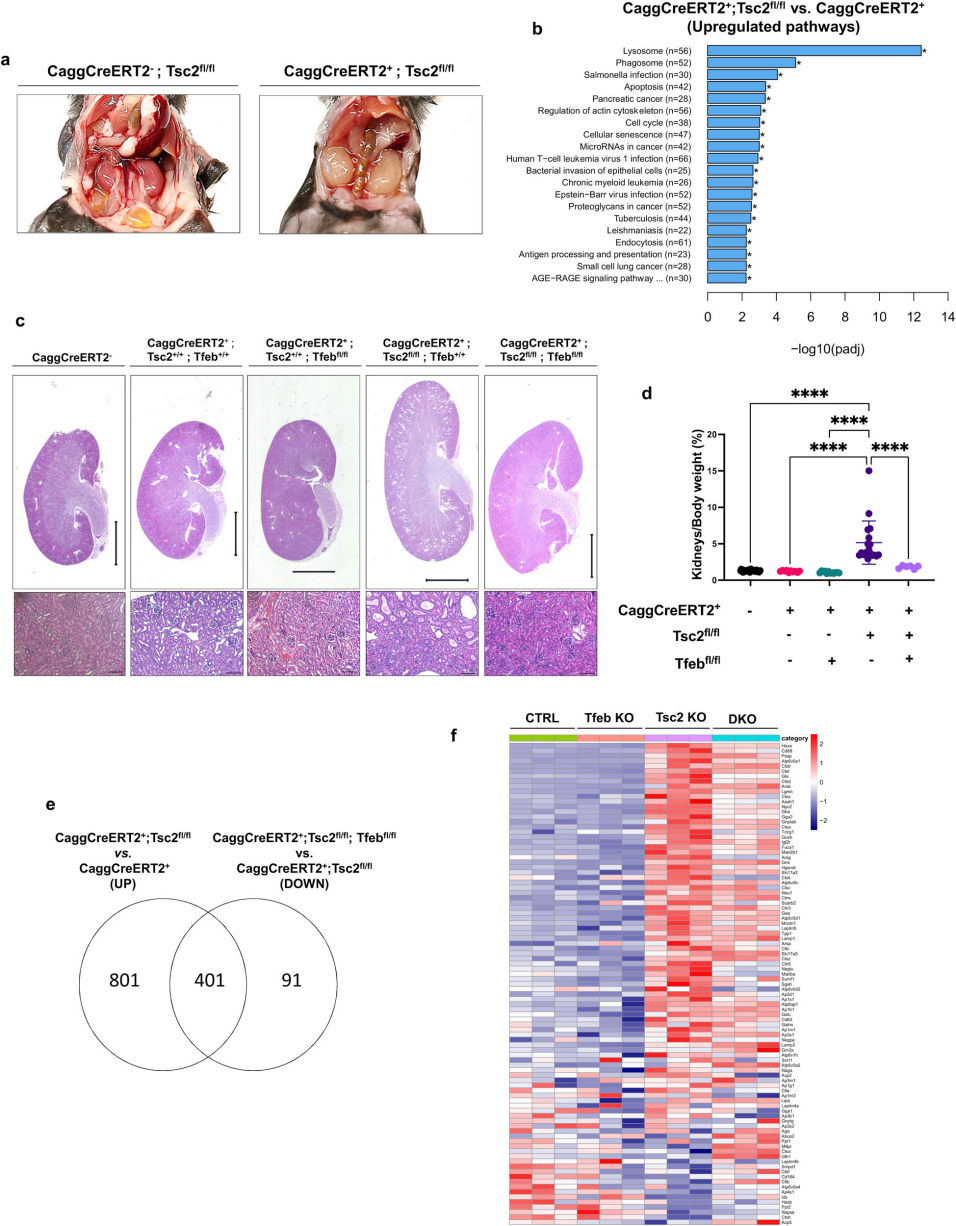
TFEB knockout rescues renal pathology in a global inducible mouse model of TSC. **a** Abdominal cavity images of tamoxifen-treated *CaggCreERT2*^*-*^*; Tsc2*^*fl/fl*^ (Ctrl) and *CaggCreERT2*^*+*^*; Tsc2*^*fl/fl*^ (*Tsc2* KO) mice at P30. **b** KEGG enrichment analysis of upregulated pathways in tamoxifen-induced *CaggCreERT2*^*+*^*; Tsc2*^*fl/fl*^ (*Tsc2* KO) mouse kidneys vs control (*CaggCreERT2*^*+*^). **c** Low (top) and high (bottom) magnification images of H&E stained kidneys of the indicated genotypes. Low magnification scale bar = 2 mm, high magnification scale bar = 100 μm. **d** Kidneys to body weight ratios of *Cre*-negative or *Cre*-expressing male and female tamoxifen-induced mice with loss of *Tsc2 (Tsc2*^*fl/fl*^*), Tfeb (Tfeb*^*fl/fl*^*)* or both *(Tsc2*^*fl/fl*^*; Tfeb*^*fl/fl*^*)* compared to control mice *(Tsc2*^*+/+*^*; Tfeb*^*+/+*^*)* at P30 (*Cre*-negative mice, *n* = 35; Cre-expressing mice, *n* = 10; *Cre*-expressing *Tfeb*^*fl/f*^ mice, *n* = 12; *Cre*-expressing *Tsc2*^*fl/fl*^ mice, *n* = 18; *Cre*-expressing *Tsc2*^*fl/fl*^*; Tfeb*^*fl/fl*^ mice, *n* = 6). **e** Venn diagram showing overlap of statistically significant genes increased by *Tsc2* deficiency and decreased by combined *Tsc2* and *Tfeb* deficiency in the *CaggCreERT2*^*+*^ kidneys using RNA sequencing at P30. **f** Heatmap of KEGG lysosome gene expression in *CaggCreERT2*^*+*^ mouse kidneys with loss of *Tsc2, Tfeb* or both (DKO) at P30. Data are presented as mean ± SD. Statistical analyses were performed using one-way ANOVA, ****p < 0.0001. Source data are provided as a Source data file.

We crossed whole-body tamoxifen-inducible Cre transgenic mice *(CaggCreERT2*^*+*^*)* with *Tsc2*^*fl/fl*^ mice to generate an inducible *Tsc2* knockout mouse line *(CaggCreERT2*^*+*^*; Tsc2*^*fl/fl*^*)*. Tamoxifen was administered to the lactating mothers for 3 days beginning at post-natal day 1 (P1) resulting in *Tsc2* inactivation in the mouse progeny via lactation. At P30, *CaggCreERT2*^*+*^*; Tsc2*^*fl/fl*^ mice were smaller in size (Supplementary Fig. 1a), with enlarged, pale kidneys (Fig. 1a) relative to corresponding tamoxifen-treated control mice (*CaggCreERT2*^*-*^ mice). Kyoto Encyclopedia of Genes and Genomes (KEGG) pathway analysis following RNA sequencing of *CaggCreERT2*^*+*^*; Tsc2*^*fl/fl*^ relative to control *CaggCreERT2*^*+*^*; Tsc2*^*+/+*^ kidneys (both groups exposed to tamoxifen) identified the lysosomal and phagosomal pathways as the most upregulated (Fig. 1b), suggesting a possible role of TFEB and/or TFE3, which are master regulators of lysosomal biogenesis^17,18^.

To evaluate the contribution of TFEB to the phenotype in this new model, we crossed the *CaggCreERT2*^*+*^*; Tsc2*^*fl/fl*^ mice with *Tfeb*^*fl/fl*^ mice to generate a *CaggCreERT2*^*+*^*; Tsc2*^*fl/fl*^*; Tfeb*^*fl/fl*^ inducible double knockout (DKO) line. *Tsc2* and *Tfeb* mRNA levels in whole kidney lysates were decreased as expected, and levels of *Gpnmb*, a well-known target of TFE3 and TFEB^33^ and histological marker of TSC2^35^, were increased about 800-fold in the *Tsc2* KO kidneys, and almost completely normalized in DKO mice (Supplementary Fig. 1b).

Strikingly, genetic inactivation of *Tfeb* rescued the kidney pathology of the *CaggCreERT2*^*+*^*; Tsc2*^*fl/fl*^ mice (Fig. 1c), decreased the kidneys to body weight ratio (Fig. 1d), normalized body weight (Supplementary Fig. 1c) and significantly corrected the transcriptional alterations associated with *Tsc2* depletion (Fig. 1e), including the induction of lysosomal gene expression (Fig. 1f and Supplementary Dataset 1). Fifty percent of the *Tsc2* KO mice had died by 40 days while all the DKO mice were alive at 80 days (Supplementary Fig. 1d). Interestingly, inducible total body knockout of *Tfeb* did not induce any apparent phenotype at the analyzed timepoints.

### TFEB knockout rescues renal pathology and lethality in kidney-specific Tsc2-knockout mice

**Fig. 2:**
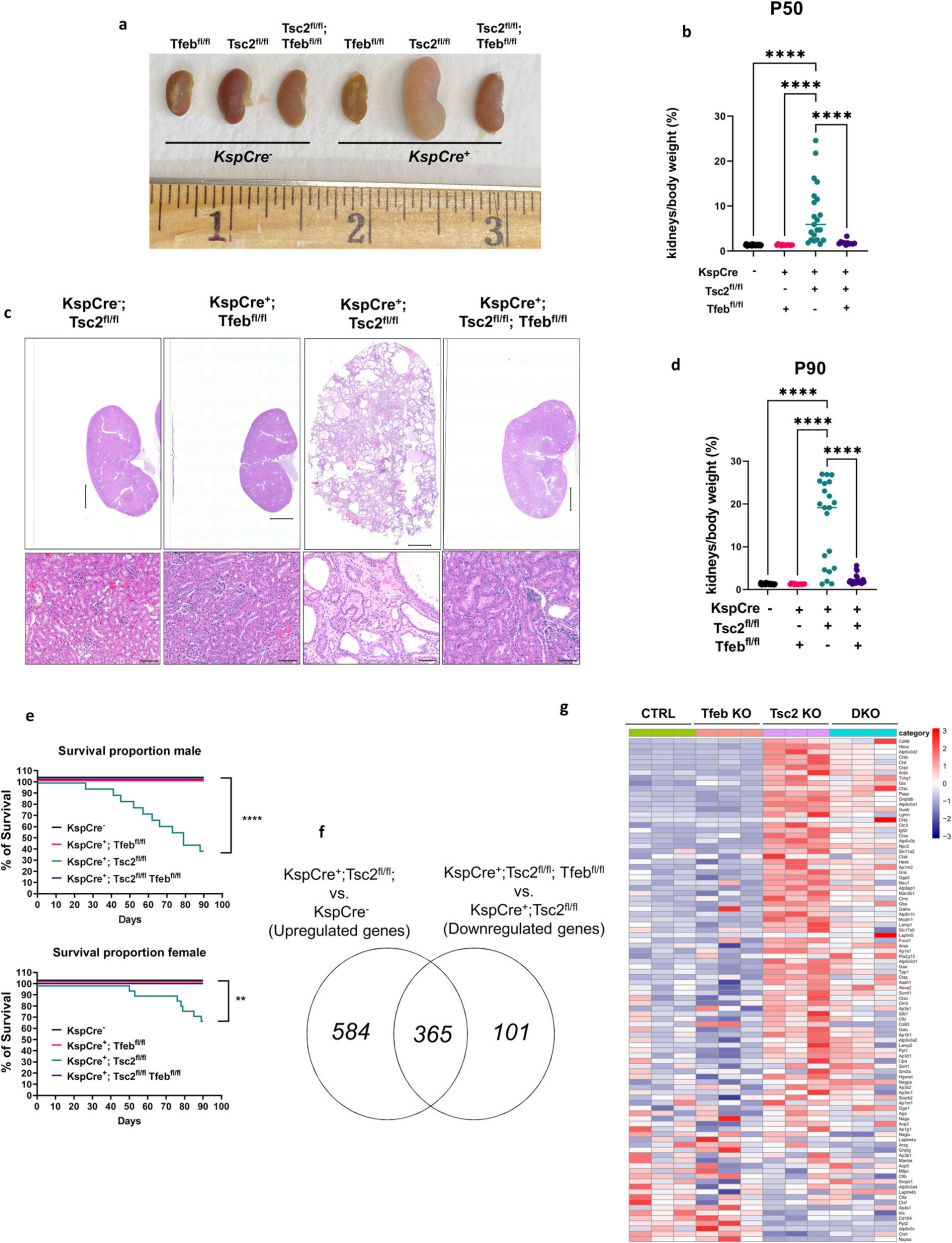
TFEB knockout rescues renal pathology and lethality in kidney-specific *Tsc2*-knockout mice. **a** Representative gross images of kidneys from the indicated genotypes at postnatal day 50 (P50). **b** Kidneys to body weight ratio of the indicated genotypes at P50 (*Cre*-negative mice, *n* = 41; *Cre*-expressing *Tfeb*^*fl/f*^ mice, *n* = 14; *Cre*-expressing *Tsc2*^*fl/fl*^ mice, *n* = 21; *Cre*-expressing *Tsc2*^*fl/fl*^*; Tfeb*^*fl/fl*^ mice, *n* = 11). **c** Low (top) and high (bottom) magnification images of H&E stained kidneys from the indicated genotypes at P90. Low magnification scale bar = 2 mm, high magnification scale bar = 100 μm. **d** Kidneys to body weight ratio of the indicated genotypes at P90 (*Cre*-negative mice, *n* = 45; *Cre*-expressing *Tfeb*^*fl/f*^ mice, *n* = 15; *Cre*-expressing *Tsc2*^*fl/fl*^ mice, *n* = 21; *Cre*-expressing *Tsc2*^*fl/fl*^*; Tfeb*^*fl/fl*^ mice, *n* = 25). **e** Kaplan–Meier curve showing survival of male (*p* = 0.0000000194) and female mice (*p* = 0.0017) in (**d**). **f** Venn diagram showing overlap of statistically significant genes increased by *Tsc2* inactivation and decreased by combined *Tsc2* and *Tfeb* inactivation in the *KspCre*^*+*^ kidneys using RNA sequencing at P50. **g** Heatmap of KEGG Lysosome gene expression in mouse kidneys with loss of *Tsc2, Tfeb* or both (DKO) at P50. Graphs are presented as mean ± SD. Statistical analyses were performed using one-way ANOVA, **p < 0.01, ****p < 0.0001. Source data are provided as a Source data file.

To specifically address the impact of *Tfeb* in *Tsc2*-deficient kidney epithelium, we next generated a second TSC mouse model by crossing kidney-specific cadherin16 Cre (*KspCre*^*+*^) mice with *Tsc2*^*fl/fl*^ mice, generating a *KspCre*^*+*^*; Tsc2*^*fl/fl*^ line. *Tsc2* and *Tfeb* mRNA levels in whole kidney lysates were decreased, as expected (Supplementary Fig. 2a). These mice were phenotypically normal at birth, but with kidney pathology evident as early as P21 (Supplementary Fig. 2b). At P50, the *KspCre*^*+*^*; Tsc2*^*fl/fl*^ mice have enlarged, pale kidneys (Fig. 2a), with numerous cysts in the renal cortex (Supplementary Fig. 2b), decreased body weight (Supplementary Fig. 2c), elevated kidneys to body weight ratio (Fig. 2b), and a 2-fold increase in blood urea nitrogen (BUN), indicating impaired kidney function (Supplementary Fig. 2d). The renal phenotype was even more severe at P90 (Fig. 2c, d and Supplementary Fig. 2e). At P90, 40% of the male *Tsc2* knockout mice, and 70% of the female *Tsc2* knockout mice were alive (Fig. 2e). TFEB was primarily localized in the nucleus of the cyst lining cells from kidneys of *KspCre*^*+*^*; Tsc2*^*fl/fl*^ mice (Supplementary Fig. 3a). Consistent with this nuclear localization of TFEB in *KspCre*^*+*^*; Tsc2*^*fl/fl*^ mice, lysosomal number was increased in cyst lining cells relative to normal adjacent tubular cells or to renal tubular cells of control mice, as assessed by transmission electron microscopy (TEM) (Supplementary Fig. 3b). Similar to our whole-body inducible *CaggCreERT2*^*+*^*; Tsc2*^*fl/fl*^ model, lysosome pathway was the most upregulated in *KspCre*^*+*^*; Tsc2*^*fl/fl*^ mice by RNA-seq (Supplementary Fig. 3c). Genetic inactivation of *Tfeb* was sufficient to normalize histology (Supplementary Fig. 2b), the body weight (Supplementary Fig. 2c, e), survival (Fig. 2e), and renal function (Supplementary Fig. 2d) of kidney-specific *Tsc2* KO, and significantly corrected the transcriptional alterations (Fig. 2f), the induction of lysosomal genes in *Tsc2* null kidneys relative to control kidneys (Fig. 2g and Supplementary Dataset 1), and the number of lysosomes (Supplementary Fig. 3b). These in vivo studies clearly indicate that TFEB plays a fundamental role in murine TSC-associated renal pathology and suggest that pharmacological inhibition of its activity may represent a valuable therapeutic strategy, especially since mice with knockout of *Tfeb* alone appear to be healthy.

### Pharmacologic and genetic inhibition of mTORC1 increases the phosphorylation and decreases the nuclear localization of TFEB and TFE3 in *TSC*-deficient cells

**Fig. 3:**
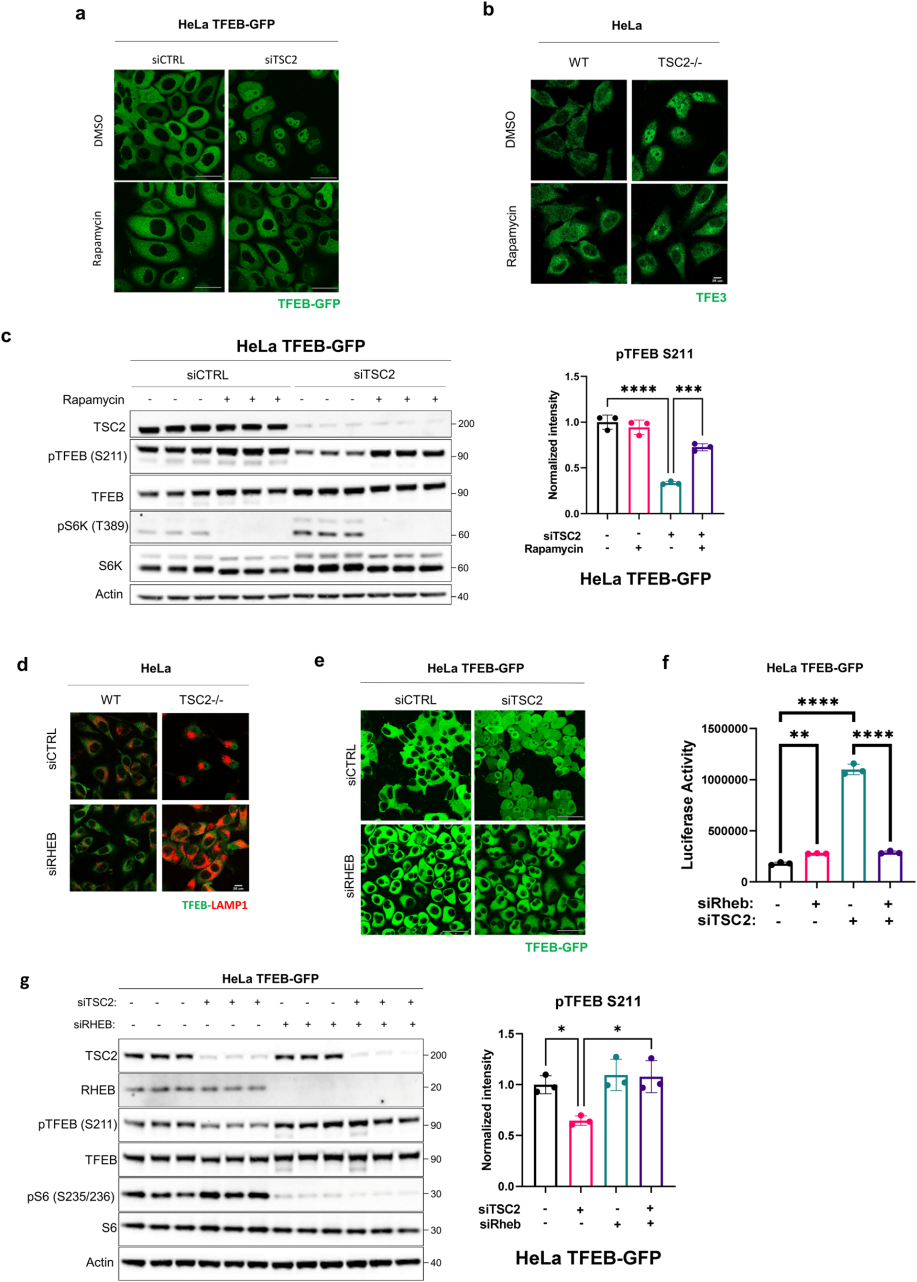
Pharmacologic and genetic inhibition of mTORC1 increases the phosphorylation and decreases the nuclear localization of TFEB and TFE3 in *TSC*-deficient cells. **a** HeLa TFEB-GFP cells were transfected with *Ctrl* or *TSC2* siRNA for 72 h, then treated with DMSO or 20 nM Rapamycin for 24 h and visualized with confocal live imaging. Scale bar = 50 μm. Cytoplasmic/nuclear localization of TFEB-GFP is quantified in Supplementary Fig. 4a. **b** HeLa cells with control or *TSC2* knockout by Crispr-Cas9 were treated with DMSO or 50 nM Rapamycin for 2 h, stained for TFE3 and visualized with confocal microscopy. Scale bar = 25 μm. Cytoplasmic/nuclear localization of TFE3 is quantified in Supplementary Fig. 4b. **c** Immunoblotting of HeLa TFEB-GFP cells treated as in a (*n* = 3 independent biological replicates per condition). Blot was analyzed by staining with the indicated antibodies, phospho-TFEB (S211) density relative to total TFEB is quantified on the right. **d** HeLa cells with control or *TSC2* knockout by Crispr-Cas9 were transfected with *Ctrl* or *RHEB* siRNA for 72 h, stained for TFEB and LAMP1 and visualized with confocal microscopy. Scale bar = 25 μm. Cytoplasmic/nuclear localization of TFEB is quantified in Supplementary Fig. 4e. **e** HeLa TFEB-GFP cells expressing *Ctrl* or *TSC2* siRNA were co-transfected with *Ctrl* or *RHEB* siRNA for 72 h and analyzed with confocal live imaging. Scale bar = 50 μm. Cytoplasmic/nuclear localization of TFEB-GFP is quantified in Supplementary Fig. 4f. **f** Luciferase activity of HeLa TFEB-GFP cells stably expressing the GPNMB luciferase reporter and transfected with indicated siRNAs for 72 h (*n* = 3 biological replicates per condition). **g** Representative immunoblotting of HeLa TFEB-GFP cells treated as in (**e**) (*n* = 3 independent biological replicates per condition). Blot was analyzed by staining with the indicated antibodies, phospho-TFEB (S211) density relative to total TFEB is quantified on the right. Scale bars = 50 μm. Data are presented as mean ± SD. Statistical analyses were performed using one-way ANOVA, *p < 0.05, **p < 0.01, ***p < 0.001, ****p < 0.0001. Source data are provided as a Source data file.

As noted earlier, mTORC1 phosphorylates TFEB at S211, inducing 14-3-3 binding and cytoplasmic localization, yet in *TSC1*-deficient and *TSC2*-deficient cells, TFEB and TFE3 are paradoxically nuclear despite high mTORC1 activity^25–27^. The use of catalytic inhibitors of mTOR, such as Torin1, promotes TFEB/TFE3 de-phosphorylation and nuclear translocation, whereas Rapamycin, a widely used allosteric inhibitor of mTORC1, does not affect TFEB or TFE3 phosphorylation in most cell types^18,23,24,27^. Rapamycin (Sirolimus) and Everolimus are FDA-approved for the treatment of TSC-associated disease in the brain, kidney, lung, and skin^36–39^. We found that Rapamycin treatment of *TSC2*-deficient HeLa TFEB-GFP cells and *TSC2*-deficient HeLa cells surprisingly increased TFEB and TFE3 cytosolic localization (Fig. 3a, b, Supplementary Fig. 4c and quantified in Supplementary Fig. 4a, b). Equally unexpectedly, Rapamycin treatment decreased phosphorylation of the canonical mTORC1 substrate S6K, but increased TFEB phosphorylation at Serine 211 (S211) (Fig. 3c), an mTORC1 site. Similar results with Rapamycin treatment were observed in TFEB-GFP HeLa cells silenced for *TSC1* (Supplementary Fig. 4d) and in *TSC2*-deficient HEK293T cells expressing TFEB-GFP (Supplementary Fig. 5a). Silencing of Rheb mimicked Rapamycin treatment, having no effect on the localization of TFEB in control cells, but markedly increasing the cytoplasmic localization of TFEB in *TSC2*-deficient HeLa cells and HeLa TFEB-GFP cells (Fig. 3d, e and quantified in Supplementary Fig. 4e, f). Luciferase assay experiments confirmed that TFEB transcriptional activity was significantly reduced upon silencing of *Rheb* in *TSC2*-deficient HeLa cells overexpressing TFEB-GFP (Fig. 3f). Rheb siRNA also increased the phosphorylation of TFEB at S211 in HeLa TFEB-GFP (Fig. 3g) and HEK293T cells expressing *TSC2* siRNA (Supplementary Fig. 5b), similarly to treatment with Rapamycin.

In contrast to the results with Rapamycin, treatment with the mTOR catalytic inhibitor Torin1 resulted in TFEB dephosphorylation and nuclear accumulation in control cells and decreased TFEB phosphorylation at S211 in *TSC2*-depleted HeLa TFEB-GFP cells (Supplementary Fig. 6a and b). Consistent with these results and with Fig. 3, the expression of the TFEB targets RagC and FLCN was markedly increased in the *TSC2*-deficient HeLa-TFEB-GFP cells and decreased by Rapamycin but not Torin1 treatment, whereas in control cells was markedly increased by Torin1 treatment but not Rapamycin (Supplementary Fig. 6b and c).

### The effect of Rapamycin on TFEB in TSC2-deficient cells is mediated by the Rag GTPases

**Fig. 4:**
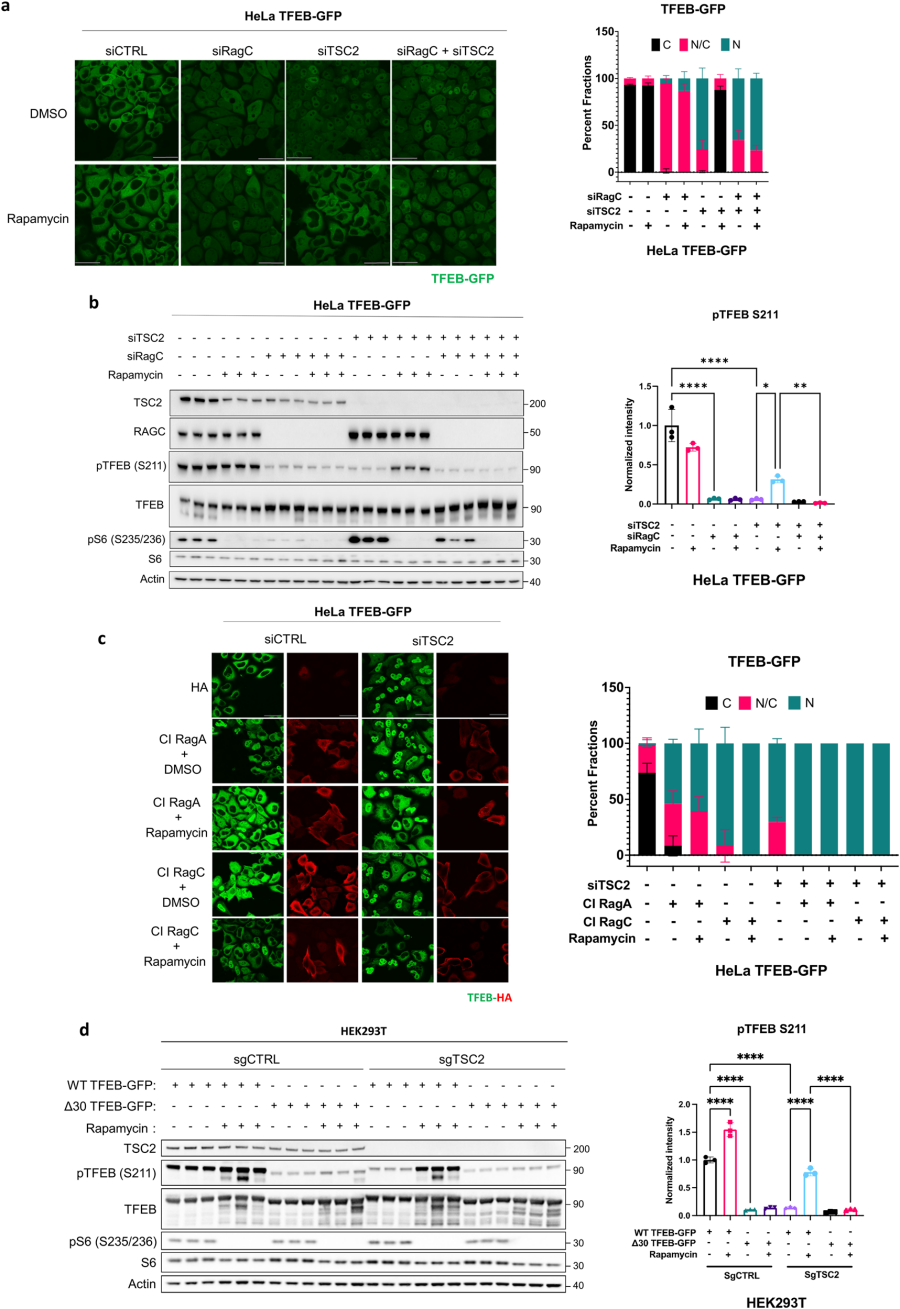
The effect of Rapamycin on TFEB in *TSC2*-deficient cells is mediated by the Rag GTPases. **a** HeLa TFEB-GFP cells transfected with *Ctrl*, *TSC2* and/or *RagC* siRNA for 72 h were treated with DMSO or 20 nM Rapamycin for 24 h and analyzed by confocal live imaging. Scale bar = 50 μm. Cytoplasmic/nuclear localization of TFEB-GFP is quantified on the right. C: cytoplasmic, N/C: nuclear/cytoplasmic, N: nuclear (*n* ≥ 130 cells quantified on 3 independent images for each condition). **b** Representative immunoblotting of HeLa TFEB-GFP cells treated as in (**a**). Blot was analyzed by staining with the indicated antibodies, phospho-TFEB (S211) density relative to total TFEB is quantified on the right (*n* = 3 independent biological replicates per condition). **c** Immunofluorescent analysis of HeLa TFEB-GFP cells after *Ctrl* and *TSC2* siRNA downregulation for 72 h, transfection with constitutively inactive (CI) RagA (pRK5 HA-RagA T21N) or RagC (pRK5 HA-RagC Q120L) for 48 h, and treatment with DMSO or 20 nM Rapamycin for 24 h. Scale bar = 50 μm. Cytoplasmic/nuclear localization of TFEB-GFP is quantified on the right. C: cytoplasmic, N/C: nuclear/cytoplasmic, N: nuclear (*n* ≥ 6 cells quantified on 3 independent images for each condition). **d** HEK293T cells with Crispr-Cas9 inactivation of TSC2 (sg*TSC2*) or control cells (sg*Ctrl*), expressing wild type (WT) TFEB-GFP or mutant TFEB-GFP without the first 30 amino acids (delta30). Cells were treated with DMSO or 20 nM Rapamycin for 24 h, phospho-TFEB (S211) density relative to total TFEB is quantified on the right (*n* = 3 independent biological replicates per condition). Scale bar = 50 μm. Data are presented as mean ± SD. Statistical analyses were performed using one-way ANOVA, *p < 0.05, **p < 0.01, ****p < 0.0001. Source data are provided as a Source data file.

Rapamycin treatment of HeLa TFEB-GFP cells in which *RagC* and *TSC2* were knocked down did not result in relocalization of TFEB to the cytoplasm or increased S211 phosphorylation of TFEB, indicating that the effects of Rapamycin in TSC are RagC dependent (Fig. 4a, b). Overexpression of inactive forms of RagA (T21N-GDP) and RagC (Q120L-GTP) (Fig. 4c) also prevented the Rapamycin-induced relocalization of TFEB in *TSC2*-deficient cells, confirming the Rag-dependence of Rapamycin’s effects. Similarly, Rapamycin treatment of *TSC2*-deficient HEK293T cells overexpressing the delta30 mutant of TFEB (lacking the N-terminal 30 amino acids which mediate binding to the Rags) failed to increase S211 phosphorylation on TFEB (Fig. 4d). Collectively, these data indicate that pharmacologic (Rapamycin) inhibition of mTORC1 paradoxically increases mTORC1-dependent phosphorylation of TFEB at S211 in a Rag-dependent manner.

### The effect of Rapamycin in TSC is mediated by TFEB

**Fig. 5:**
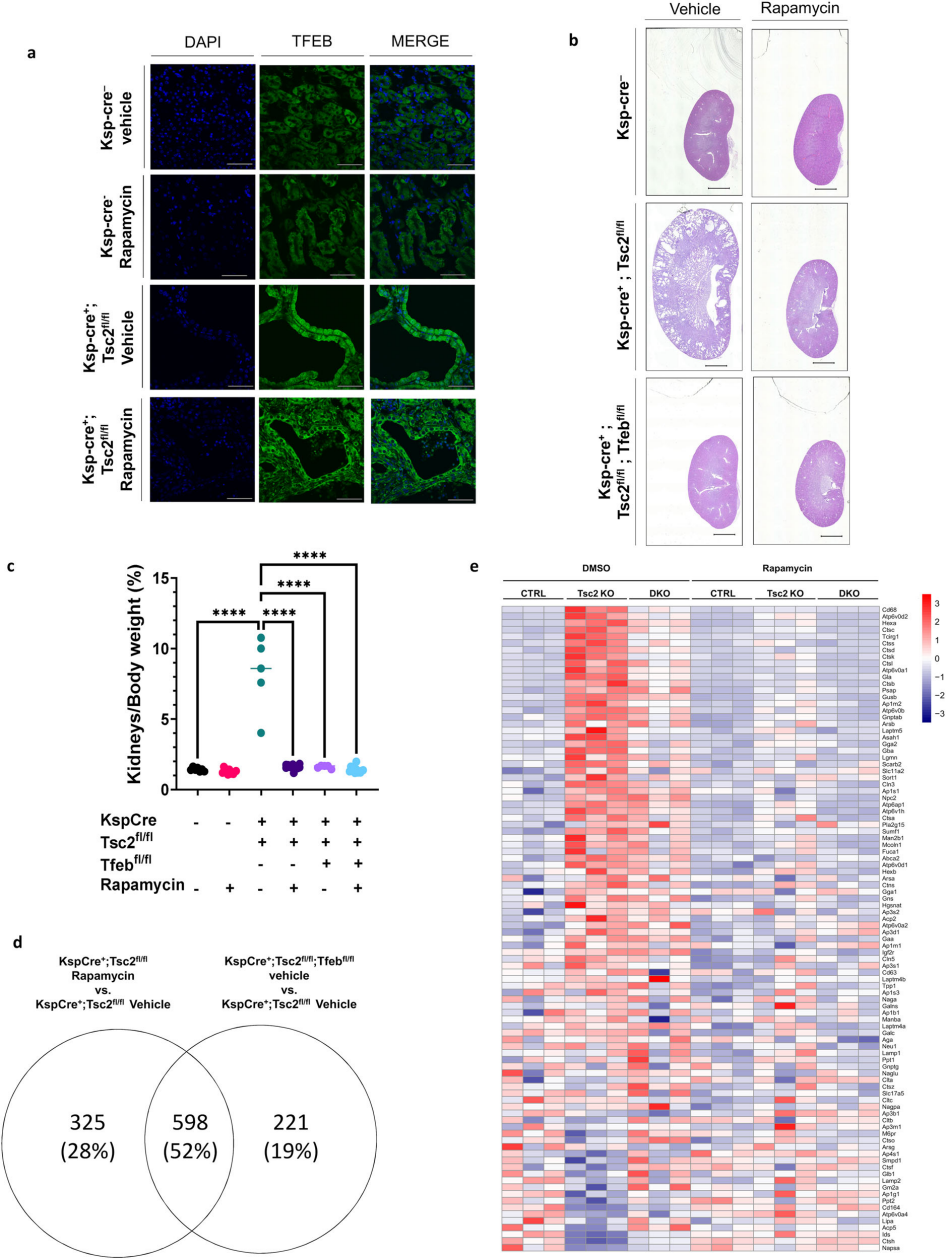
The effect of Rapamycin in TSC is mediated by TFEB. **a** Immunofluorescent analysis of TFEB localization in kidney sections from the indicated genotypes treated with vehicle control or Rapamycin (1 mg/kg 3 times a week) for 1 week beginning at P43 and harvested at P50 (total of 3 injections). Scale bar = 50 μm. **b** Representative images of H&E-stained kidneys from the indicated genotypes at P50 treated with vehicle control or Rapamycin (1 mg/kg 3 times a week) every other day for 25 days (total of 11 injections), scale bar = 2 mm. (*Cre*-negative vehicle-treated mice, *n* = 45; *Cre*-negative Rapamycin treated mice, *n* = 11; *Cre*-expressing *Tsc2*^*fl/fl*^ vehicle-treated mice, *n* = 5; *Cre*-expressing *Tsc2*^*fl/fl*^ Rapamycin treated mice, *n* = 11; *Cre*-expressing *Tsc2*^*fl/fl*^*; Tfeb*^*fl/fl*^ vehicle-treated mice, *n* = 5; *Cre*-expressing *Tsc2*^*fl/fl*^*; Tfeb*^*fl/fl*^ Rapamycin treated mice, *n* = 12). **c** Kidneys to body weight ratios from the indicated genotypes treated as in (**b**). **d** Venn diagram showing overlap of statistically significant genes increased by *Tsc2* KO and decreased by Rapamycin treatment (as in b) or decreased by combined *Tsc2/Tfeb* KO in *KspCre*^*+*^ kidneys using RNA sequencing. **e** Heatmap of KEGG Lysosome gene expression in kidneys of Ctrl, *Tsc2* KO, or *Tsc2/Tfeb* double KO mice treated with vehicle control or Rapamycin (as in (**b**)). Data are presented as mean ± SD. Statistical analyses were performed using one-way ANOVA, ****p < 0.0001. Source data are provided as a Source data file.

To determine how Rapamycin treatment impacts TFEB subcellular localization and activity in vivo, kidney specific *Tsc2* KO mice (*KspCre*^*+*^*; Tsc2*^*fl/fl*^) were treated with vehicle or Rapamycin (1 mg/kg/3 times a week) for one week (a total of 3 injections) starting at P43 and kidneys collected at P50. TFEB was primarily localized in the nucleus of the cyst lining cells from kidneys of *Tsc2* KO (*KspCre*^*+*^*; Tsc2*^*fl/fl*^) mice and was relocalized outside of the nucleus upon this short-term Rapamycin treatment (Fig. 5a), consistent with our in vitro data (Fig. 3). Importantly, longer Rapamycin treatment (1 mg/kg/3 times per week, starting at P25, for a total of 11 injections) of kidney-specific *Tsc2* KO mice and *Tsc2/Tfeb* DKO (*KspCre*^*+*^*; Tsc2*^*fl/fl*^*;Tfeb*^*fl/fl*^) mice revealed that Rapamycin treatment and *Tfeb* inactivation were equally effective in correcting kidney size, kidney pathology, and kidneys to body weight ratio of *Tsc2* KO mice (Fig. 5b, c). Interestingly, gene expression analysis indicated that >50% of the genes that are significantly upregulated in the *Tsc2-*deficient kidneys were downregulated by both Rapamycin treatment and *Tfeb* inactivation, including most of lysosomal genes (Fig. 5d, e and Supplementary Dataset 1). Collectively, these data indicate that inhibition of TFEB significantly contributes to the beneficial effect of Rapamycin in TSC.

### TFEB regulates mTORC1 activation in TSC

**Fig. 6:**
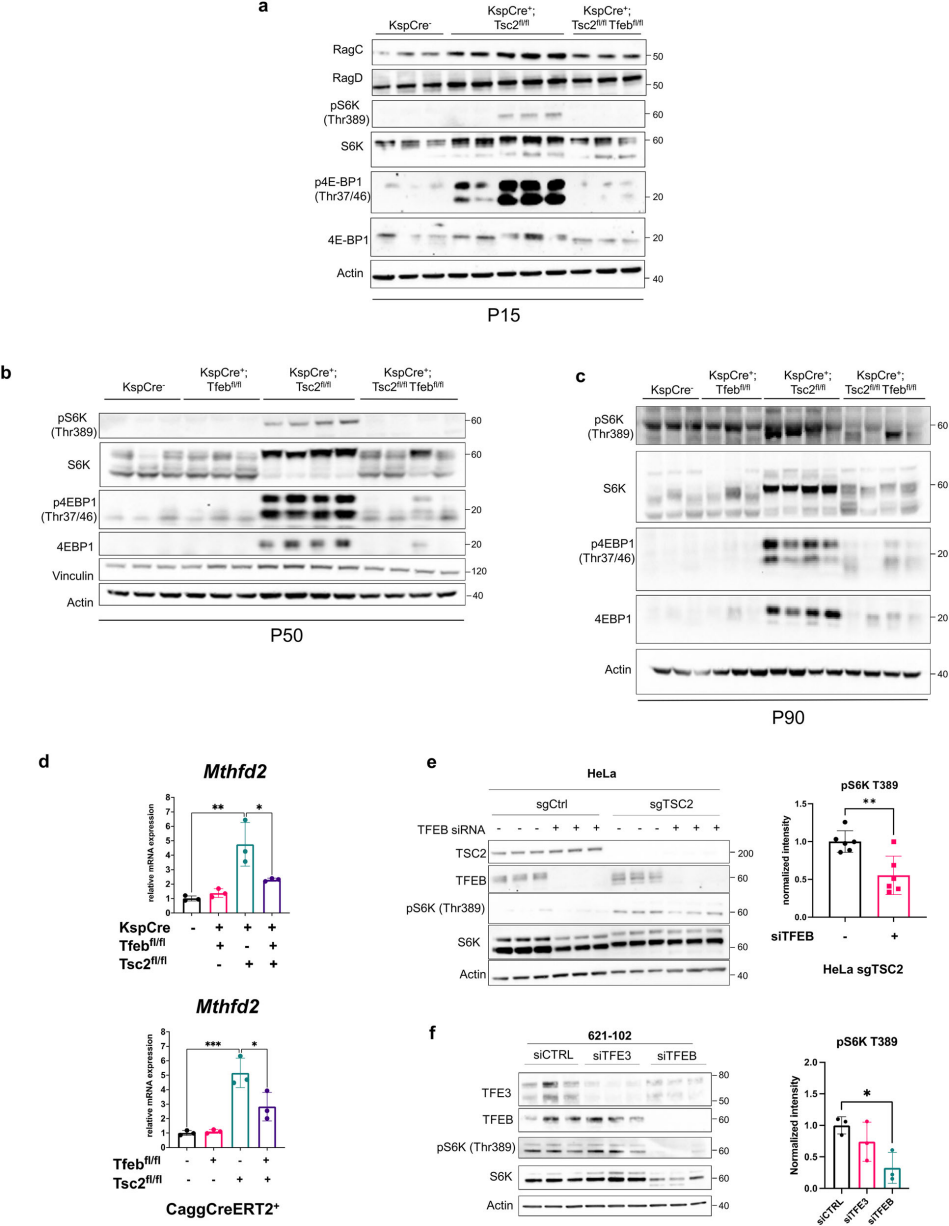
TFEB regulates mTORC1 activation in TSC. **a** Immunoblot analyses of whole kidney lysates from *KSP-Cre* mice of the indicated genotypes at P15 (*n* = 3 independent Cre-negative and Cre-expressing *Tsc2*^*fl/fl*^*; Tfeb*^*fl/fl*^ animals, *n* = 5 independent *Cre*-expressing *Tsc2*^*fl/fl*^ animals). Densitometry analysis of relative protein expression of RagC and RagD in immunoblots from a is quantified in Supplementary Fig. 7a. **b** Immunoblot analyses of whole kidney lysates from *KSP-Cre* mice (fasted overnight prior to harvest) of the indicated genotypes at P50 (*n* = 3 or 4 independent animals per genotype). **c** Immunoblot analyses of whole kidney lysates from mice of the indicated genotypes at P90 (*n* = 3 or 4 independent animals per genotype). **d**, qRT-PCR analysis of *Mthfd2* expression in *KspCre* and *CaggCreERT2*^*+*^ mice with loss of *Tsc2*, *Tfeb* or both at P50 and P30 respectively (*n* = 3 independent animals). **e** Representative immunoblotting of HeLa cells with control or *TSC2* knockout by Crispr-Cas9 transfected with Ctrl or *TFEB* siRNA for 72 h (*n* = 6 independent biological replicates). Phospho-S6K (T389) density relative to total S6K is quantified on the right (*p* = 0.0036). **f** Immunoblotting of *TSC2*-deficient 621-102 cells transfected with Ctrl, *TFEB* or *TFE3* siRNA for 72 h (*n* = 3 independent biological replicates). Phospho-S6K (T389) density relative to total S6K is quantified on the right (*p* = 0.00142). Data are presented as mean ± SD. Statistical analyses were performed using one-way ANOVA, *p < 0.05, **p < 0.01, ***p < 0.001. Source data are provided as a Source data file.

To determine whether increased activity of TFEB contributes to mTORC1 hyperactivation in TSC, we monitored mTORC1 signaling in kidney-specific *Tsc2* KO and in *Tsc2/Tfeb* DKO mouse kidney lysates. As expected, phospho-levels of the mTORC1 substrates 4EBP1 and S6K were elevated in the *Tsc2* KO kidney lysates. Strikingly, the hyperphosphorylation of 4EBP1 (Thr37/46) and S6K (Thr389) was normalized upon genetic inactivation of *Tfeb* in *KspCre*^*+*^*; Tsc2*^*fl/fl*^ mice at P15 (before the onset of cysts) (Fig. 6a), at P50 (Fig. 6b) and at P90 (Fig. 6c). In addition, total levels of several key mTOR regulators were upregulated in the *Tsc2* KO kidney lysates and decreased in the DKO kidneys, including RagC and RagD at P15 (Fig. 6a and quantified in Supplementary Fig. 7a), RagC, RagD and Lamtor1 at P50 and P90 (Supplementary Fig. 7b, c). Levels of phospho-4EBP1 and total 4EBP1 were also elevated in the *Cagg-CreERT2*^*+*^ *Tsc2* KO kidneys and decreased in the DKO kidneys (Supplementary Fig. 7d).

To further examine whether TFEB regulates mTORC1 activity in TSC, we analyzed expression of *Mthfd2* (methylenetetrahydrofolate dehydrogenase 2) in the mouse kidneys. *Mthfd2* was shown by Brendan Manning’s laboratory to be strongly associated with mTORC1 activity^40^. *Mthfd2* was increased 4.5–5 fold in both *Ksp-Cre* and *CAGG-Cre* kidneys (Fig. 6d) and decreased by more than 50% in the double KO kidneys, consistent with a TFEB-dependent mTORC1 hyperactivation.

To determine whether similar effects of TFEB on mTORC1 activity occur in vitro, TFEB was downregulated in HeLa cells with Crispr knockout of *TSC2* and phosphorylation of S6K at Thr389, the mTORC1 site, was monitored. In *TSC2*-deficient cells, siRNA silencing of *TFEB* decreased the ratio of phospho-S6K to total S6K by about 50% (Fig. 6e). Similarly, in *TSC2*-deficient 621-102 cells^41^, which are derived from a human angiomyolipoma, downregulation of *TFEB* decreased the phospho-S6K/total S6K ratio by 60%. Downregulation of *TFE3* in these cells did not significantly impact phospho-S6K (Fig. 6f). Together with the in vivo results, these data support a key role for TFEB in upregulating mTORC1 activity in TSC.

## Discussion

Our data reveal for the first time that TFEB is the critical driver of renal disease in two mouse models of TSC. Both total body tamoxifen-inducible *Cagg-Cre* and kidney epithelium-specific *KSP-Cre* models of *Tsc2* inactivation exhibit significant cystic kidney disease burden, decreased body weight and decreased survival. Remarkably, inactivation of *Tfeb* almost completely prevented TSC-associated kidney cystic disease, decreased mTORC1 signaling and partially “normalized” TSC-dependent transcriptional changes in both mouse models.

Several lines of complementary evidence support TFEB-dependent regulation of mTORC1 activity in TSC. First, levels of phospho-4EBP1 and phospho-S6K are increased in *Tsc2* KO kidneys and decreased in the *Tsc2/Tfeb* double KO kidneys. Similarly, levels of *Mthfd2*, an established marker of mTORC1 activity, are increased 4.5-5-fold in *Tsc2* KO kidneys and decreased in the *Tsc2/Tfeb* double KO kidneys. Lysosomal proteins critical for mTOR activity (RagC, RagD, and LAMTOR1, as shown in Fig. 6 and Supplementary Fig. 7) are upregulated in the *Tsc2* KO kidneys and their levels are decreased in the double KO kidneys. Our results complement those of Bonucci et al.^42^. who found that knockout of S6K partially reversed kidney pathology in a model of TSC. We hypothesize that knockout of TFEB has a stronger impact than knockout of S6K because knockout of TFEB decreases the phosphorylation of multiple mTORC1 substrates, including S6K.

Lysosome number is also increased in *Tsc2*-deficient kidneys in a TFEB-dependent manner. Lysosomes are critical signaling hubs for mTOR signaling and one possibility is that increased lysosomal mass in *TSC*-deficient cells provides more docking surface for mTORC1. We observed consistent but milder effects of TFEB downregulation on mTORC1 activity in our *TSC*-deficient cell lines. The reasons for the differences between the in vivo and in vitro results remain unclear, in vivo factors such as the immune microenvironment (which is increasingly recognized as a component of tumorigenesis in TSC^43,44^) or nutrient/oxygen availability may be involved.

The mouse models of *Tsc2* inactivation presented here do not replicate the typical genetic mechanisms of kidney disease in human TSC, in which a germline “first hit” mutation is followed by a stochastic “second hit” mutation, but they clearly indicate that in the mouse kidney, TFEB plays a critical role in TSC-associated pathology. The other members of the TFEB family (TFE3, MITF, and TFEC) may impact TSC-associated kidney disease and other manifestations of TSC, via similar mechanisms. Indeed, MITF is considered a hallmark gene of lymphangioleiomyomatosis (LAM) and angiomyolipomas in TSC^44,45^, based on upregulation of genes that are known transcriptional targets of MITF/TFE3/TFEB/TFEC including MelanA and Cathepsin K^46,47^.

Our data presented here support and contribute to the understanding of a distinction between the canonical substrates of mTORC1 (including S6K and 4EBP1) and the non-canonical substrates (including TFEB and TFE3)^20,21,48^. In TSC, the mTORC1 canonical substrates are hyperphosphorylated and the non-canonical substrates are, surprisingly, hypophosphorylated, leading to increased nuclear TFEB/TFE3. Recently, a cryo electron microscopy structure of the lysosomal TFEB-mTORC1-Rag-Ragulator megacomplex was published, revealing the structural basis of the differentiation between canonical and non-canonical mTOR substrates^49^. Whether the clinical manifestations of human TSC are primarily the result of hyperactivity of the canonical substrates or hyperactivity of the non-canonical substrates remains an open question, but it is clear that in the murine kidney, the non-canonical substrate TFEB predominates^50^.

Currently, the only approved therapy for TSC-associated renal disease is mTORC1 inhibition with Everolimus (a Rapamycin derivative), which can be associated with serious and potentially life-threatening adverse effects^36,51^, often requiring dose reductions or treatment breaks. We found that Rapamycin treatment and Rheb knockdown, both of which inhibit mTORC1-dependent phosphorylation of its canonical substrates, paradoxically increase phosphorylation of TFEB at S211, an mTORC1 site, leading to decreased nuclear TFEB and decreased TFEB activity in *TSC*-deficient cells. The mechanisms underlying the unexpected Rapamycin and Rheb downregulation-induced nuclear exclusion and increase in TFEB phosphorylation remain unclear. We hypothesize that in TSC, mTORC1 inhibition may impair the FLCN-RagC axis and/or lead to a conformational change in mTORC1, resulting in increased phosphorylation and nuclear exclusion of TFEB.

Our data suggest that the therapeutic benefit of Rapamycin in TSC occurs at least partially via TFEB, since mTORC1 inhibition moves TFEB out of the nucleus in *TSC2*-deficient cells, in vitro and in vivo, and more than 50% of the genes that are downregulated by Rapamycin treatment of *Tsc2*-deficient kidneys are also decreased by knockout of TFEB. Future experiments comparing Rapamycin and Torin1 treatment could help to further dissect the canonical versus non-canonical substrate predominance in our mouse models of TSC. In *TSC*-deficient cells, we have shown that Rapamycin inhibits both canonical and non-canonical substrates, by moving TFEB/TFE3 out of the nucleus. In contrast, Torin1 inhibits only the canonical substrates, since TFEB remains nuclear upon Torin1 treatment.

The evidence that kidney-specific knockout of TFEB has no detectable effects on kidney physiology (Figs. 1, 2) suggests that targeting TFEB could represent an alternative and potentially safer therapeutic strategy for TSC-associated renal disease, and potentially for other TSC-associated manifestations.

In summary, we have discovered that TFEB plays a fundamental role in the development of kidney disease downstream of *Tsc2* knockdown in two distinct mouse models of TSC. The TSC complex is an established negative modulator of mTORC1 signaling, and mTORC1 hyperactivation is the widely accepted hallmark of TSC-associated tumors. For 20 years, it has been believed that hyperactivation of Rheb upon loss of *TSC1* or *TSC2* is solely responsible for mTORC1 hyperactivity in TSC. Our data support a paradigm-shifting model in which TFEB, through the Rag GTPases, is also required for mTORC1 activation in *TSC2*-deficient cell lines and genetically engineered mouse models of TSC.

## Methods

All performed experiments complied with Brigham and Women’s Hospital Chemical and Biological safety and Animal Care and Use Committee regulations.

### Cell culture

HeLa cells and HEK293T cells were purchased from ATCC. Hela TFEB-GFP cells were developed by the Shawn Ferguson lab^23^. 621-102 and 621-103 cells were developed and characterized previously^41^. All cells were grown in Dulbecco’s Modified Eagle Medium (DMEM, Gibco/Thermo Fisher Scientific, USA) supplemented with 10% fetal bovine serum with 1% penicillin/streptomycin. Rapamycin and Torin1 were purchased from LC Laboratories.

### Generation of HeLa TSC2 CRISPR knock-out line

HeLa cells (ATCC CCL-2) with knockout of the *TSC2* gene were generated by using the CRISPR/Cas9 system. The gRNA for the *TSC2* gene (CCAACGAAGACCTTCACGAA) was selected using the http://crispor.tefor.net/crispor.py online tool. HeLa cells were electroporated with the gRNA and the CAS9 protein (*HiFi Cas9 Nuclease V3 IDT cat# 1081060*) using the Amaxa system (*kit Cat No VCA-1003 from Lonza)*. Cells were FACS sorted into 96 well plates to obtain single cell-derived colonies carrying the INDEL mutations. Upon genomic DNA extraction, the genomic sequence containing the targeted region were amplified by PCR reaction with the specific primers: hTSC2up=TGCTGATCCTGTGGCTTTTG and hTSC2down=CCAGAGAAACCTCCAACCCA. PCR products were analyzed by DNA Sanger sequencing and clones carrying the homozygous deletion introducing a PSC were selected, expanded, and validated by western blot. *Tsc2* KO HeLa cells used in Fig. 6e were described previously^25^.

### Generation of HEK293T TSC2 CRISPR knock-out line

Non-targeting control gRNA and *TSC2* gRNA plasmids were designed using the pRP_gRNA_Cas9 plasmid backbone from Vector Builder. The expression of small guide RNAs and mCherry-tagged human codon-optimized Cas9 from Streptococcus pyogenes was driven by U6 and modified chicken beta-actin (CBh) promoters respectively. Both non-targeting control and *TSC2* CRISPR plasmids included 2 small guide RNAs (GTGTAGTTCGACCATTCGTG and GTTCAGGATCACGTTACCGC for non-targeting control; TCCTTGCGATGTACTCGTCG and GACCCGGTCGTTACTAGGCC for *TSC2*). Plasmids were transiently expressed in HEK293T cells using Fugene 6 (Promega, USA) for 48 h and single-cell sorted for mCherry on 96-well plates. Single clones were isolated and *TSC2* knockout was verified by immunoblotting.

### siRNA transfection

siRNA transfections were performed using Lipofectamine RNAiMax (ThermoFisher, 13778150). The following Silencer Select siRNA reagents from ThermoFisher were used: *Tsc2* (s502596), *Tsc2* (s533034), *Tsc1* (s526384), *Rheb* (s12019), *RRagC* (s34476), *Tfeb* (s15497), *Tfe3* (s14030), and non-targeting control (4390844). in Fig. 3d, siRNA for *Rheb* (#14267) was from Cell Signaling and the non-targeting siRNA Pool (D-001810-10-05) was from Dharmacon.

### Live imaging

Hela-TFEB-GFP cells were plated onto 35 mm glass bottom dishes (Mattek, USA). Images were captured using an Olympus Fluoview FV10i confocal microscope.

### Immunofluorescence

Cells were plated onto 35 mm glass bottom dishes, fixed with 2% paraformaldehyde (PFA) for 15 min, permeabilized with 0.1% Triton X-100 for 5 min and washed three times with PBS. Cells were then blocked for 30 min in 1% BSA and incubated with primary antibodies: anti-GFP (1:1000) (ab13970, Abcam, USA) and anti-HA-tag (1:1000) (3724, Cell Signaling Technology, USA) in blocking buffer (1% BSA) for 1 h. Cells were then washed with PBS and stained with secondary antibody (1:1000 dilution) anti-Rabbit Alexa Fluor Green 488 and Red 568 (Life Technologies/ThermoFisher) in blocking buffer and kept in the dark for 1 h. DAPI (4’, 6-diamidino-2-phenylindole) (Sigma-Aldrich, USA) was used to visualize nuclei. Cells were washed again with PBS and mounted with VECTASHIELD Antifade Mounting Medium (Vector Laboratories, USA). Images were captured using an Olympus Fluoview FV10i confocal microscope. Confocal images were analyzed for percentage of nuclear localization using ImageJ as previously described^25^ or by manual quantification in a blinded fashion.

To detect endogenous TFEB, TFE3, and LAMP1 the following antibodies were used: TFEB (1:200) (Cell Signaling cat. 4240), TFE3 (1:200) (Cell Signaling cat. 14779) and LAMP1 (H4A3) (1:500) (Santa Cruz cat. 20011). Cells were fixed in 4% PFA for 15 min and permeabilized with 0.1% Triton X-100 for 5 min. Blocking was performed with 3% bovine serum albumin in PBS + 0.02% saponin for 1 h at RT. Cells were incubated with the indicated primary antibodies overnight and then with secondary antibodies for 1 h (AlexaFluor 488, 1:500, Thermo Fisher). For confocal imaging, the samples were examined under a Zeiss LSM 800 confocal microscope. Optical sections were obtained under a ×63 immersion objective.

### Protein extraction and western blot analysis

Cells were washed with ice-cold PBS, scraped, and lysed on ice with 1X RIPA buffer. Lysates were normalized by concentration and resolved on 4-12% Bis-Tris gel. The following antibodies were used at 1:1000 dilution and purchased from Cell Signaling Technology unless otherwise indicated: b-actin (Sigma-Aldrich, A5316), b-actin (1:5000) (Sigma-Aldrich, A2066), b-actin (4970), Vinculin (4650), GFP (1:5000) (Abcam, ab13970), phosphorylated S6 ribosomal protein (pS6 Ser 235/236) (2211), total S6 ribosomal protein (TS6) (2217), TSC2 (4308), mouse-specific TFEB (32361), human-specific TFEB (37785), TFE3 (14779), FLCN (3697), phosphorylated TFEB S211 (37681), RagC (9480), RagD (Abcam, ab1877679), LAMTOR1 (8975), Rheb (13879), phosphorylated p70 S6 Kinase (pS6K Thr389) (9234), p70 S6 Kinase (2708), 4EBP1 (9644), and phosphorylated 4EBP1 (p4EBP1 Thr37/46) (2855). The relative protein expression was quantified using ImageJ software in 3 biological replicates.

### Plasmids

The following plasmids from Addgene were used: CI RagA (pRK5 HA-RagA T21N) #99711, CI RagC (pRK5 HA-RagC Q120L) #99720 and pEGFP-N1-TFEB (Addgene #38119). TFEB-GFP wild-type and delta30 mutants were developed by Dr. Shawn Ferguson^23^. The GPNMB reporter activity plasmid was previously described^25^. Plasmids were expressed using Fugene6 reagent (Promega) for immunofluorescence or Lipofectamine 3000 (Invitrogen) for immunoblotting.

### Animal studies

Animal studies were approved by the Brigham and Women’s Hospital Animal Care and Use Committee. All mice were maintained in a C57BL/6 strain background and housed in an animal facility with 12 h light/12 h dark cycle at 72 °F and 40% humidity with ad libitum access to food and water unless otherwise specified. Mice were maintained on irradiated PicoLab Rodent Diet 20 (5053). The *Tfeb*^*fl/fl*^ mice were developed by Andrea Ballabio^24^. *Tsc2*^*fl/fl*^, *CaggCreERT2*, and *KspCre (Cadh16Cre)* mice were acquired from Jackson Laboratories. Recombination in *CaggCreERT2* mice was achieved by intraperitoneal Tamoxifen injection (62.5 μg/g of body weight) in lactating mothers at day 1, 2 and 3 postpartum. Rapamycin (1 mg/kg of body weight) was administered intraperitoneally every other day from day 25 to day 50 (11 injections) or from day 43 to day 50 (3 injections). Blood for Blood Urea Nitrogen (BUN) measurements was collected from P50 mice by heart puncture. Serum was separated from the blood by centrifugation in EDTA-containing tubes (BD Microtainer) and then frozen at −80C. BUN measurements were done by VRL Animal Health Diagnostics (Maryland, USA).

### Mouse kidney immunofluorescence

Mice were perfused with 4% paraformaldehyde in PBS by cardiac puncture. Optimal Cutting Temperature compound (OCT)-embedded kidney sections were permeabilized with 0.5% Triton X-100 for 5 min and washed three times with PBS. Sections were then blocked for 30 min in 1% BSA and incubated with primary antibody TFEB (1:50 dilution) (Bethyl Laboratories, A303-673A), in blocking buffer (1% BSA) overnight. Sections were then washed three times with PBS and stained with secondary antibody (1:1000 dilution) anti-Rabbit Alexa Fluor Green 488 (Life Technologies/ThermoFisher), in blocking buffer (1% BSA) and kept in the dark for 1 h. DAPI (4′, 6-diamidino-2-phenylindole) (Sigma-Aldrich) was used to visualize nuclei. Sections were washed again three times with PBS and mounted with VECTASHIELD Antifade Mounting Medium (Vector Laboratories).

### Electron microscopy

Kidney specimens were fixed in 2% glutaraldehyde/2% paraformaldehyde in 0.1 M phosphate buffer for 3 h at 4 °C, postfixed in 1% osmium tetroxide in the same buffer solution, dehydrated in graded alcohols, and embedded in an Epon-Araldite mixture. Thin sections were stained with lead citrate and examined with a CM10 transmission electron microscope. Ten random images were utilized for lysosome quantitation.

### mRNA extraction, real-time PCR, and RNA-sequencing

mRNA from flash frozen mouse kidneys or cultured human cells was isolated using the RNeasy MiniKit (Qiagen). cDNA was generated using the high capacity reverse transcription kit (4368814, Thermo Fisher). Real-time PCR was conducted using the StepOne Plus Realtime PCR Machine (Applied Biosystems) with TaqMan Real-Time PCR Master Mix (Thermo Fisher Scientific). Gene expression was measured relative to mouse *b-actin* (4351315) or human *b-actin* (4325788). The delta delta Ct (ΔΔCt) method was used to calculate fold change differences. Human and mouse-specific TaqMan real-time PCR assays (Thermo Fisher Scientific) with optimized probe/primer sets were used for *Tfeb* (Mm00448968_m1), *Tsc2* (Mm00442004_m1), *Gpnmb* (Mm01328587_m1), *Mthfd2* (Mm00485276_m1). RNA-sequencing: library preparation and sequencing were performed by Novogene (Sacramento, CA, USA). Differentially expressed genes were defined as having FPKM values of at least 5 and adjusted *p* values of 0.05 or lower.

### Statistical analyses and reproducibility

All quantitative, normally distributed data were analyzed for statistical significance using a Student’s unpaired *t*-test, or one-way ANOVA and Tukey’s post-hoc tests when comparing more than two groups relative to a single factor, or two-way ANOVA and Sidak’s post-hoc tests when comparing more than two groups relative to more than one factor. GraphPad Prism Software (www.graphpad.com) was used. Log-rank test was used for the survival analysis.

All experiments have been repeated independently at least three times.

### Reporting summary

Further information on research design is available in the Nature Portfolio Reporting Summary linked to this article.

### Supplementary information

Supplementary Information

Peer Review File

Description of Additional Supplementary Files

Supplementary Data 1

Reporting Summary

### Source data

Source Data

## Data Availability

All RNA sequencing data generated in this study have been deposited to Gene Expression Omnibus (GEO) under accession code GSE244072 and are freely accessible. Source data are provided with this paper in a Source Data file. Uncropped western blots for data in Main and Supplementary Figures are provided in the Source Data file. Source data are provided with this paper.
