# Supplementary material for: TFEB drives mTORC1 hyperactivation and kidney disease in Tuberous Sclerosis Complex
Source: Nat Commun. 2024 Jan 9;15:406. doi: 10.1038/s41467-023-44229-4 (PMC10776564; doi:10.1038/s41467-023-44229-4)
Supplement: Supplementary file 1 — Supplementary Information [file 41467_2023_44229_MOESM1_ESM.pdf]

Supplementary Information

TFEB drives mTORC1 hyperactivation and kidney disease in Tuberous Sclerosis Complex

Supplementary Figure 1

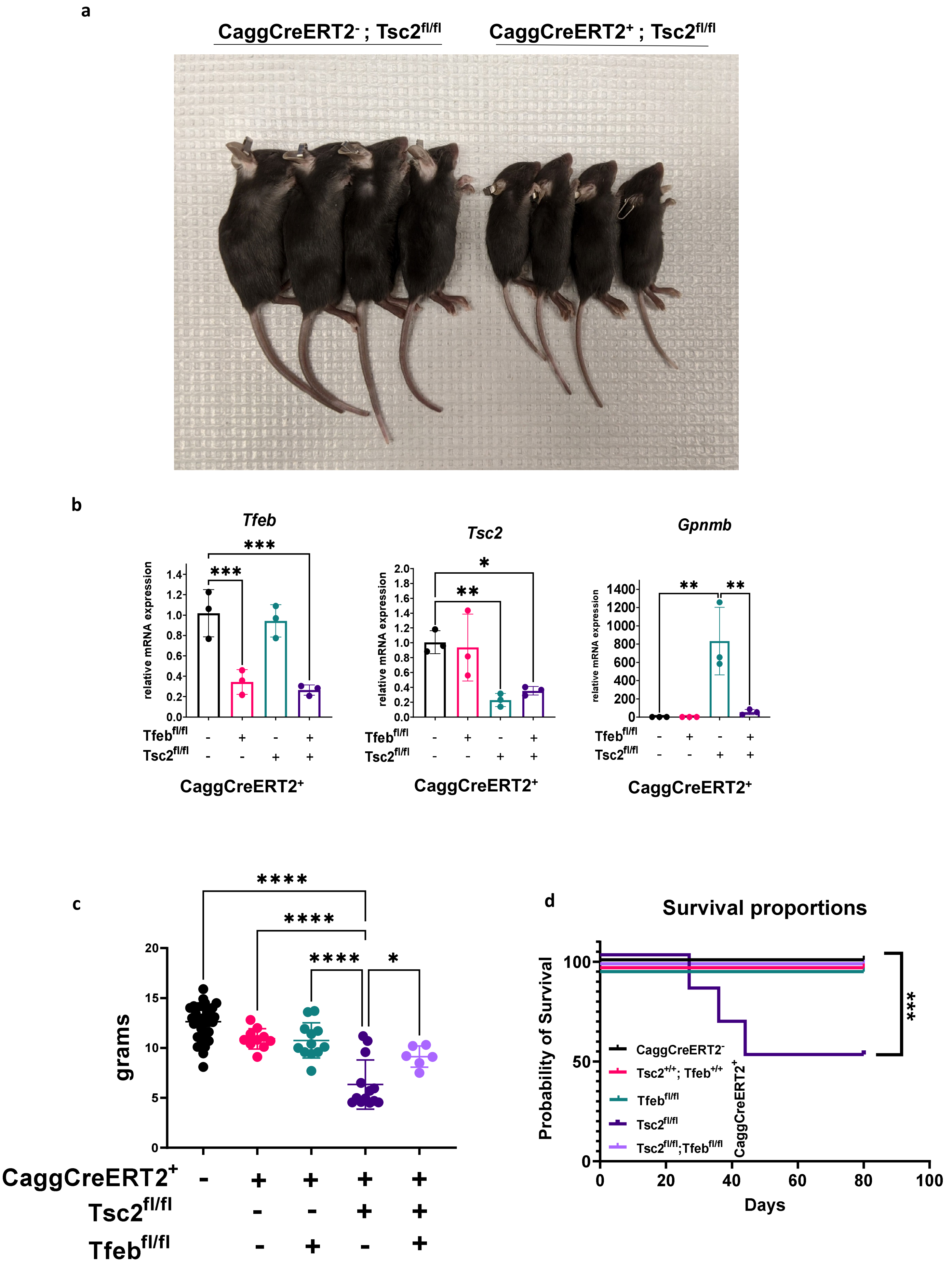

Supplementary Figure 1: TFEB knockout rescues renal pathology in a global inducible model of TSC

a, Whole-body image of tamoxifen treated *CaggCreERT2*$^{-}$*; Tsc2*$^{fl/fl}$ (Ctrl) and *CaggCreERT2*$^{+}$*; Tsc2*$^{fl/fl}$ (*Tsc2* KO) mice at P30. **b,** qRT-PCR analysis of *Tsc2*, *Tfeb* and *Gpnmb* expression in tamoxifen-induced *CaggCreERT2*$^{+}$ kidneys with loss of *Tsc2, Tfeb* or both (n = 3 independent animals). **c**, Body weight of mice from the indicated genotypes at P30 (*Cre*-negative mice, n = 35; *Cre*-expressing mice, n = 10; *Cre*-expressing *Tfeb*$^{fl/f}$ mice, n = 12; *Cre*-expressing *Tsc2*$^{fl/fl}$ mice, n = 18; *Cre*-expressing *Tsc2*$^{fl/fl}$; *Tfeb*$^{fl/fl}$ mice, n = 6). **d**, Kaplan-Meier survival curve for mice of the indicated genotypes (p = 0.0003). Data are presented as mean ± SD. Statistical analyses were performed using one-way ANOVA, *p<0.05, **p<0.01, ***p<0.001, ****p<0.0001. Source data are provided as a Source data file.

Supplementary Figure 2

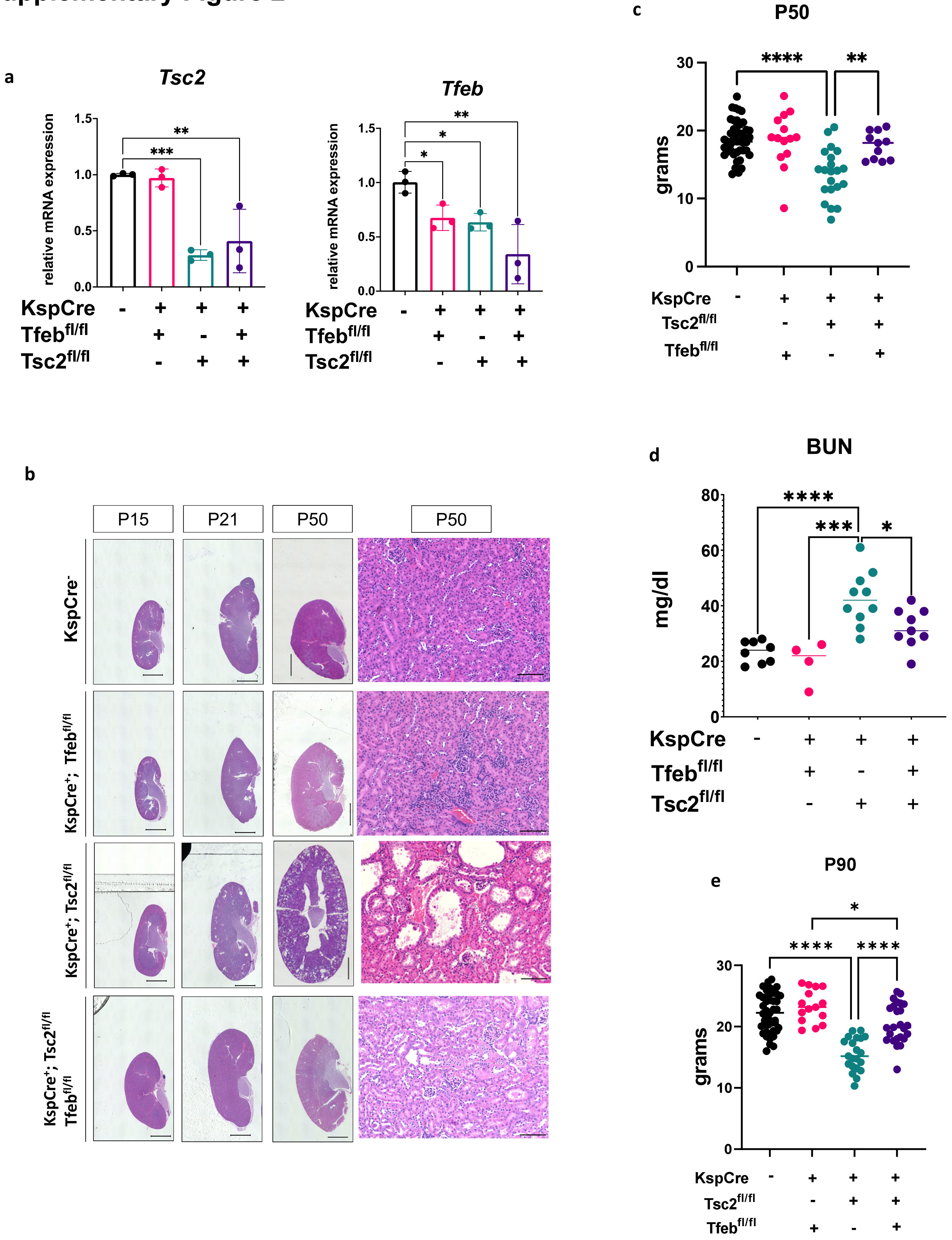

Supplementary Figure 2: TFEB knockout rescues renal function and lysosome number in kidney-specific *Tsc2* knockout mice

a, qRT-PCR analysis of *Tsc2* and *Tfeb* expression in *KspCre* mice from the indicated genotypes (n = 3 independent animals). **b**, Images of H&E-stained kidneys from the indicated genotypes at P15, P21 and P50. Scale bar = 2mm. Right column: higher magnification (20X) at P50. Scale bar = 100um. **c**, Body weight from the indicated genotypes at P50 (*Cre*-negative mice, n = 41; *Cre*-expressing *Tfeb*$^{fl/f}$ mice, n = 14; *Cre*-expressing *Tsc2*$^{fl/fl}$ mice, n = 21; *Cre*-expressing *Tsc2*$^{fl/fl}$; *Tfeb*$^{fl/fl}$ mice, n = 11). **d**, Blood urea nitrogen (BUN) levels of the indicated genotypes at P50 (*Cre*-negative mice, n = 8; *Cre*-expressing *Tfeb*$^{fl/f}$ mice, n = 4; *Cre*-expressing *Tsc2*$^{fl/fl}$ mice, n = 10; *Cre*-expressing *Tsc2*$^{fl/fl}$; *Tfeb*$^{fl/fl}$ mice, n = 9). **e**, Body weight from the indicated genotypes at P90 (*Cre*-negative mice, n = 45; *Cre*-expressing *Tfeb*$^{fl/f}$ mice, n = 15; *Cre*-expressing *Tsc2*$^{fl/fl}$ mice, n = 21; *Cre*-expressing *Tsc2*$^{fl/fl}$; *Tfeb*$^{fl/fl}$ mice, n = 25). Data are presented as mean ± SD. Statistical analyses were performed using one-way ANOVA, *$p<0.05$, **$p<0.01$, ***$p<0.001$, ****$p<0.0001$. Source data are provided as a Source data file.

Supplementary Figure 3

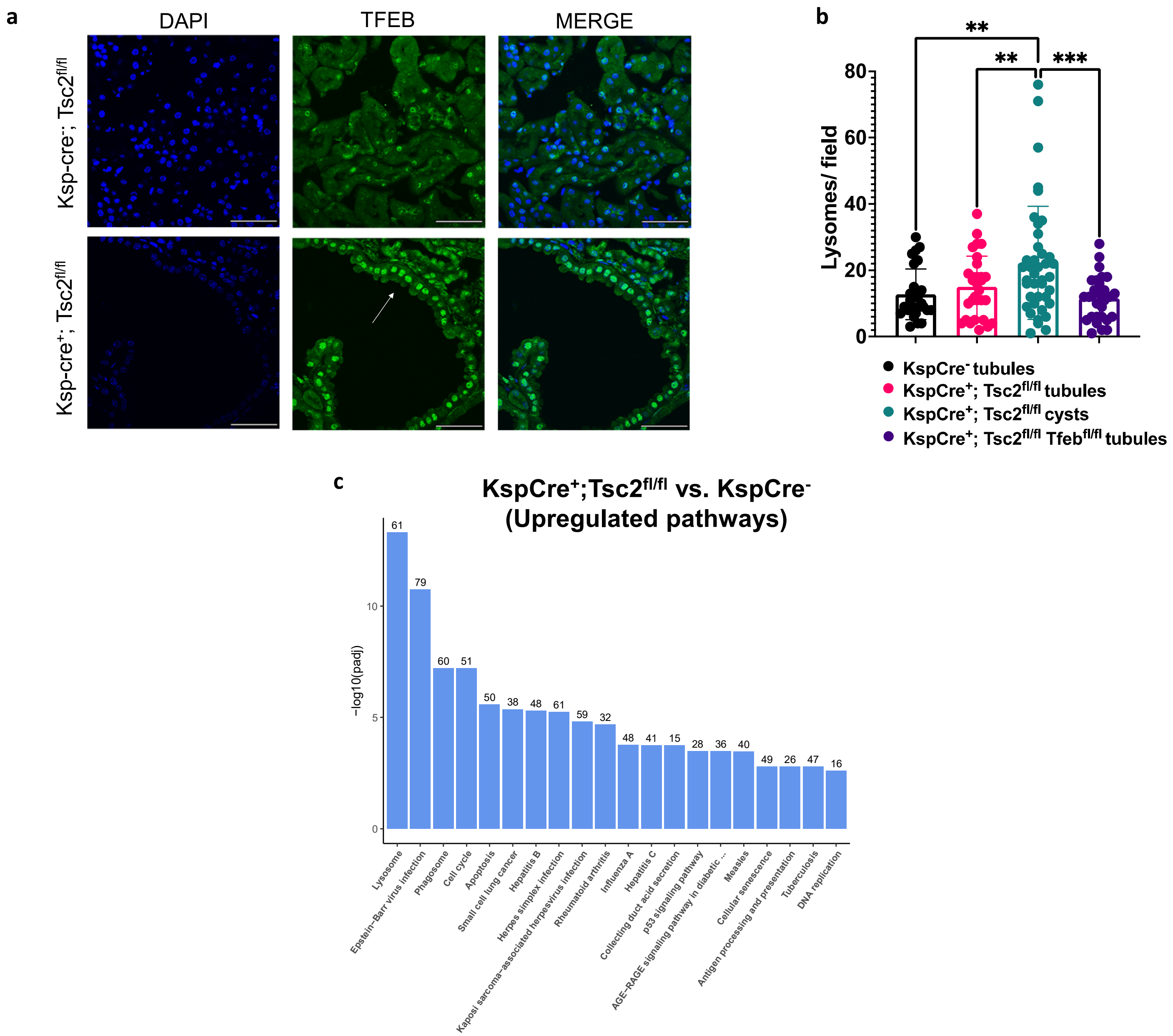

Supplementary Figure 3: TFEB knockout rescues lysosome number in kidney-specific *Tsc2* knockout mice

a, Immunofluorescent analysis of TFEB localization in kidney sections from the indicated genotypes at P50. White arrow indicates cyst lining cells with nuclear TFEB. Scale bar = 50um. **b**, Transmission electron microscopy (TEM) analysis of lysosome number in cyst lining cells in *Tsc2* KO compared with normal appearing tubule cells in *Tsc2* KO, normal tubule cells in *Tfeb* KO and normal tubule cells in *Tsc2* KO/*Tfeb* KO mice. Each dot represent the number of lysosomes/ field of observation (n ≥ 300 lysosomes quantified in 30-40 random fields in 3 independent *Cre*-negative and *Cre*-expressing *Tsc2$^{fl/fl}$; Tfeb$^{fl/fl}$* animals and 4 *Cre*-expressing *Tsc2$^{fl/fl}$* animals). **c**, KEGG enrichment analysis of upregulated pathways in *KspCre^{+}; Tsc2$^{fl/fl}$* mouse kidneys vs control (*KspCre^{-}*). Graphs are presented as mean ± SD. Statistical analyses were performed using one-way ANOVA, **p<0.01, ***p<0.001. Source data are provided as a Source data file.

Supplementary Figure 4

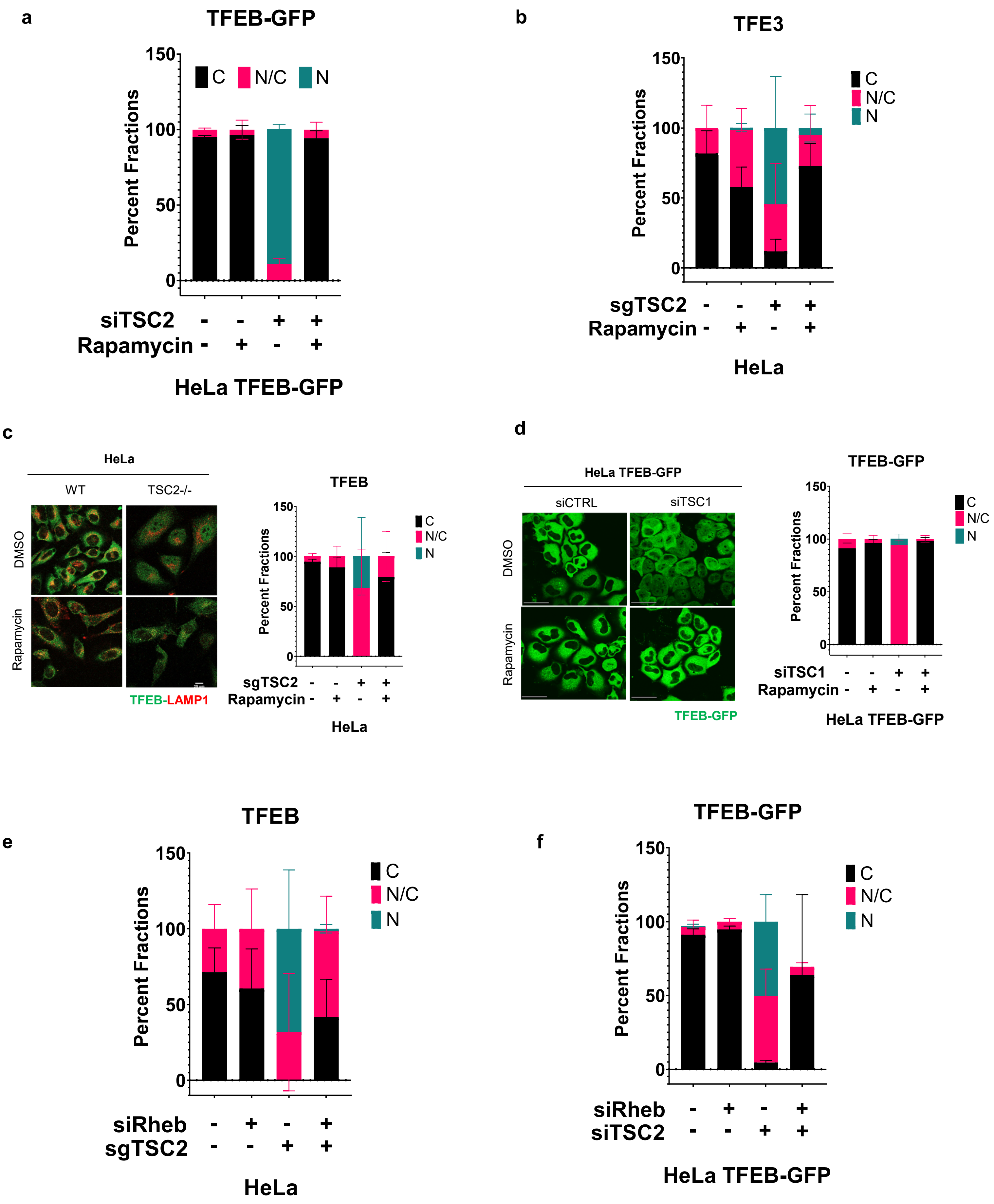

Supplementary Figure 4: Rapamycin treatment induces cytoplasmic localization in *TSC2*-deficient HeLa cells

a, Quantification of cytoplasmic/nuclear localization of TFEB-GFP for experiment in Fig. 3a ($n \geq 37$ cells quantified on 3 independent images for each condition). **b**, Quantification of cytoplasmic/nuclear localization of TFE3 for experiment in Fig. 3b ($n \geq 27$ cells quantified on 3 independent images for each condition). **c**, HeLa cells with control or *TSC2* knockout by Crispr-Cas9 were treated with DMSO or 50nM Rapamycin for 2 h, stained for TFEB and LAMP1 and visualized with confocal microscopy. Scale bar = 25um. Cytoplasmic/nuclear localization of TFEB is quantified on the right ($n \geq 27$ cells quantified on 3 independent images for each condition). **d**, HeLa TFEB-GFP cells transfected with *Ctrl* or *TSC1* siRNA for 72 h were treated with DMSO or 20 nM Rapamycin for 24 h and visualized with confocal live imaging. Scale bar = 50um. Cytoplasmic/nuclear localization of TFEB-GFP is quantified on the right ($n \geq 62$ cells quantified on 3 independent images for each condition). **e,** Quantification of cytoplasmic/nuclear localization of TFEB for experiment in Fig. 3d ($n \geq 34$ cells quantified on 3 independent images for each condition). **f,** Quantification of cytoplasmic/nuclear localization of TFEB-GFP for experiment in Fig. 3e. C: cytoplasmic, N/C: nuclear/cytoplasmic, N: nuclear ($n \geq 120$ cells quantified on 3 independent images for each condition). Data are presented as mean ± SD. Source data are provided as a Source data file.

Supplementary Figure 5

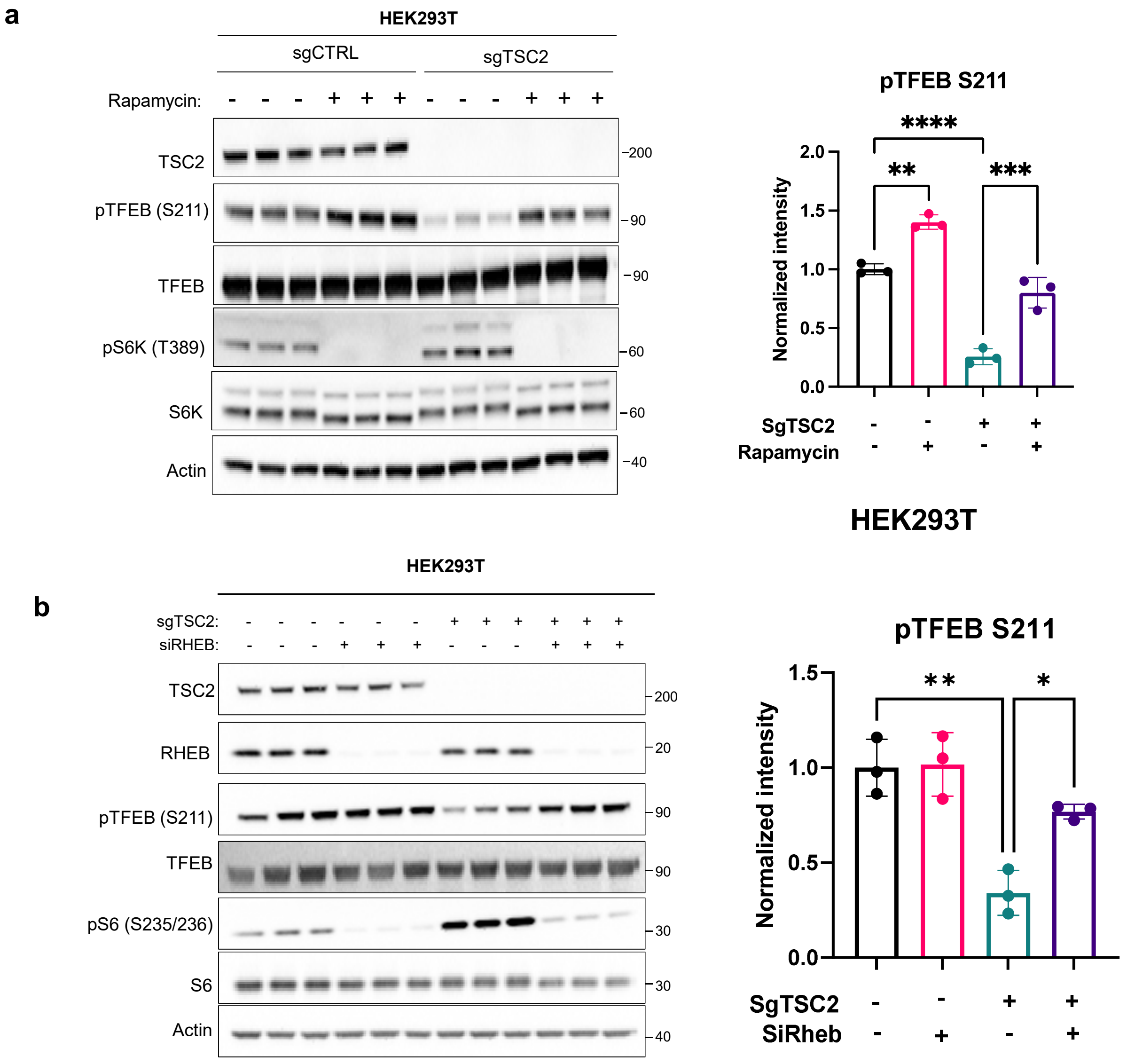

Supplementary Figure 5: Rapamycin treatment or downregulation of *RHEB* induces cytoplasmic localization in *TSC2*-deficient HEK293T cells

a, Representative immunoblotting of HEK293T cells with Crispr-Cas9 inactivation of *TSC2* (sg*TSC2*) or control cells (sg*Ctrl*) overexpressing TFEB-GFP and treated with 20nM Rapamycin or DMSO for 24 hr. Blot was analyzed by staining with the indicated antibodies, phospho-TFEB (S211) density relative to total TFEB is quantified on the right (n = 3 biological replicates). **b**, Representative immunoblotting of HEK293T cells with Crispr-Cas9 inactivation of *TSC2* (sg*TSC2*) or control cells (sg*Ctrl*) transfected with *Ctrl* or *RHEB* siRNA for 72 h. Blot was analyzed by staining with the indicated antibodies, phospho-TFEB (S211) density relative to total TFEB is quantified on the right (n = 3 biological replicates). Data are presented as mean ± SD. Statistical analyses were performed using one-way ANOVA, *p<0.05, **p<0.01, ***p<0.001, ****p<0.0001. Source data are provided as a Source data file.

Supplementary Figure 6

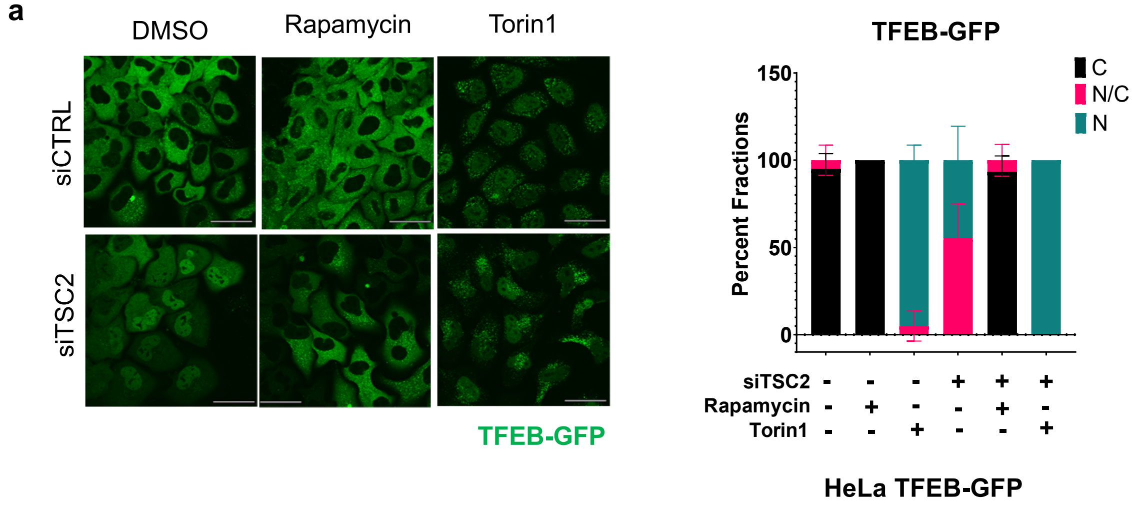

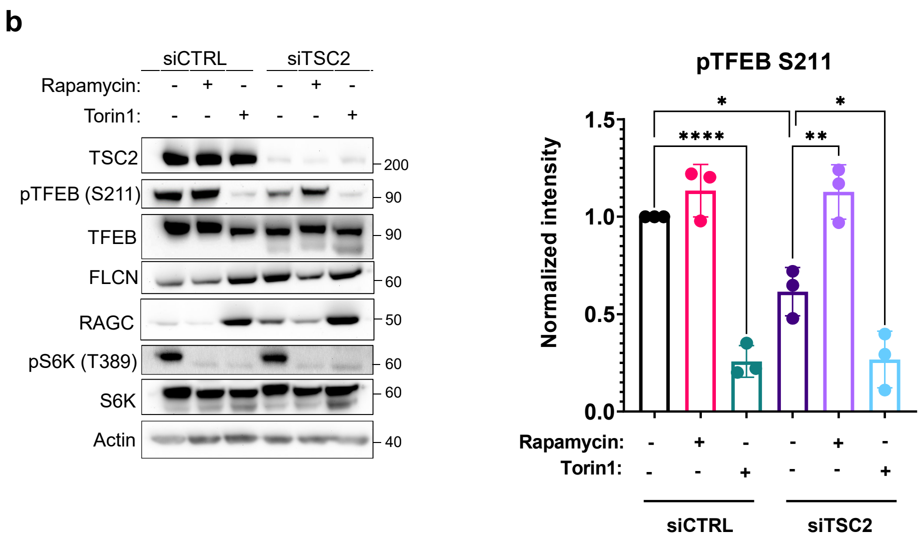

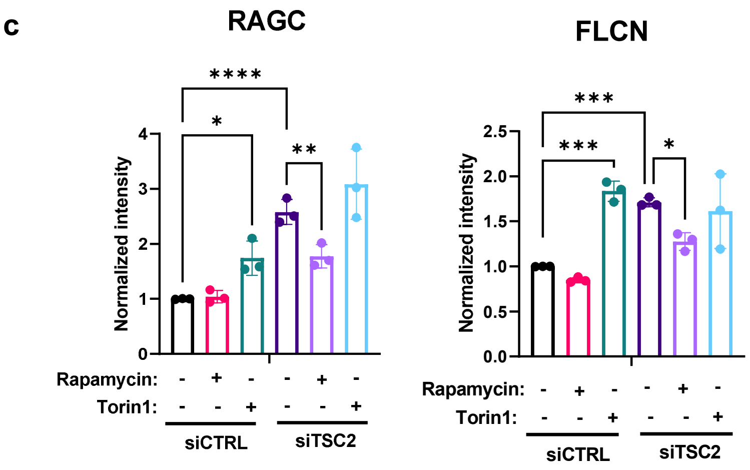

Supplementary Figure 6: Differential effects of Rapamycin and Torin1 on TFEB nuclear localization and phosphorylation in *TSC2*-deficient cells

a, HeLa TFEB-GFP cells transfected with *Ctrl* or *TSC2* siRNA for 72 h, treated with DMSO, 20 nM Rapamycin or 250nM Torin1 for 24 h and visualized with confocal live imaging. Scale bar = 50um. Cytoplasmic/nuclear localization of TFEB-GFP is quantified on the right. C: cytoplasmic, N/C: nuclear/cytoplasmic, N: nuclear (n ≥ 50 cells quantified on 3 independent images for each condition). **b,** Representative immunoblotting of HeLa TFEB-GFP treated as in a (n = 3 biological replicates per condition). Blot was analyzed by staining with the indicated antibodies. Phospho-TFEB (S211) density relative to total TFEB is quantified below. **c**, Quantification of RAGC and FLCN protein density relative to actin in HeLa TFEB-GFP treated as in b. Data are presented as mean ± SD. Statistical analyses were performed using one-way ANOVA, *p<0.05, **p<0.01, ***p<0.001, ****p<0.0001. Source data are provided as a Source data file.

Supplementary Figure 7

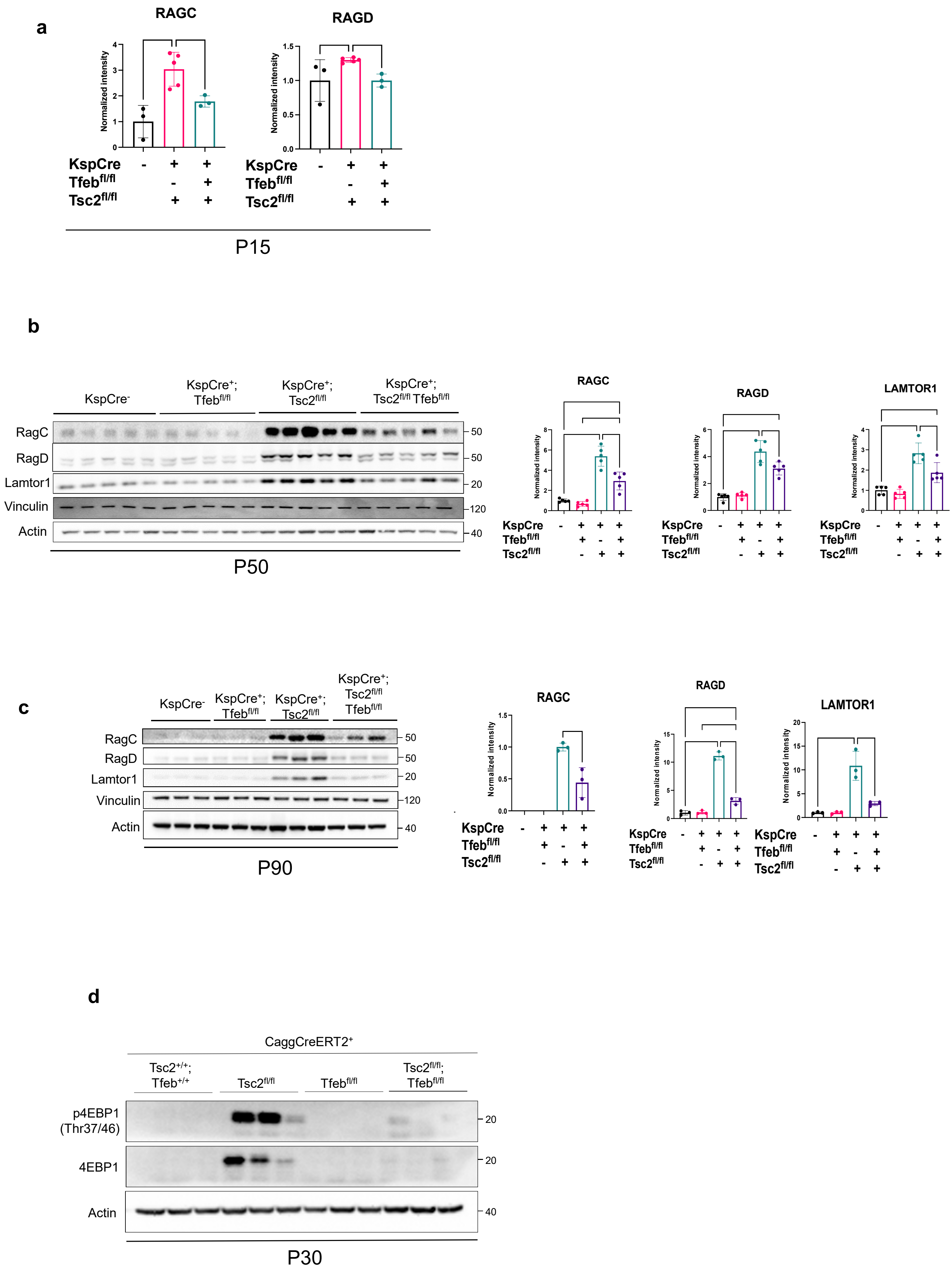

Supplementary Figure 7: TFEB regulates expression of Rag-Ragulator proteins in *TSC2*-deficient cells.

a, Densitometry analysis of relative protein expression of RagC and RagD in immunoblots in Fig. 6a. **b,** Immunoblot analysis of whole kidney lysates from *KspCre* mice of the indicated genotypes at P50 (n = 5 independent animals per genotype). RAGC, RAGD, and LAMTOR1 density relative to vinculin are quantified on the right. **c**, Immunoblot analysis of whole kidney lysates from *KspCre* mice of the indicated genotypes at P90 (n = 3 independent animals per genotype). RAGC, RAGD, and LAMTOR1 density relative to vinculin are quantified on the right. **d,** Immunoblot analysis of whole kidney lysates from *CaggCreERT2* mice of the indicated genotypes at P30 (n = 3 independent animals per genotype). Data are presented as mean ± SD. Statistical analyses were performed using one-way ANOVA, *p<0.05, **p<0.01, ***p<0.001, ****p<0.0001. Source data are provided as a Source data file.
