# Supplementary material for: TFEB drives mTORC1 hyperactivation and kidney disease in Tuberous Sclerosis Complex
Source: Nat Commun. 2024 Jan 9;15:406. doi: 10.1038/s41467-023-44229-4 (PMC10776564; doi:10.1038/s41467-023-44229-4)
Supplement: Supplementary file 3 — Description of Additional Supplementary Files [file 41467_2023_44229_MOESM3_ESM.pdf]

Description of Additional Supplementary Files

Supplementary Data 1: Expression of KEGG Lysosome pathway genes by RNA sequencing in Cagg-Cre cohort (from heatmap in Fig. 1f), KSP-Cre cohort (from heatmap in Fig. 2g) and KSP-Cre cohort with Rapamycin treatment (from heatmap in Fig. 5e).
