# Supplementary material for: TFEB drives mTORC1 hyperactivation and kidney disease in Tuberous Sclerosis Complex
Source: Nat Commun. 2024 Jan 9;15:406. doi: 10.1038/s41467-023-44229-4 (PMC10776564; doi:10.1038/s41467-023-44229-4)
Supplement: Supplementary file 5 — Reporting Summary [file 41467_2023_44229_MOESM5_ESM.pdf]

nature portfolio

Corresponding author(s): Elizabeth Petri Henske
Nicola Alesi
Andrea Ballabio

Last updated by author(s): Nov 16, 2023

Reporting Summary

Nature Portfolio wishes to improve the reproducibility of the work that we publish. This form provides structure for consistency and transparency in reporting. For further information on Nature Portfolio policies, see our Editorial Policies and the Editorial Policy Checklist.

Statistics

For all statistical analyses, confirm that the following items are present in the figure legend, table legend, main text, or Methods section.

n/a	Confirmed	
☐	☒	The exact sample size (*n*) for each experimental group/condition, given as a discrete number and unit of measurement
☐	☒	A statement on whether measurements were taken from distinct samples or whether the same sample was measured repeatedly
☐	☒	The statistical test(s) used AND whether they are one- or two-sided<br>*Only common tests should be described solely by name; describe more complex techniques in the Methods section.*
☒	☐	A description of all covariates tested
☐	☒	A description of any assumptions or corrections, such as tests of normality and adjustment for multiple comparisons
☐	☒	A full description of the statistical parameters including central tendency (e.g. means) or other basic estimates (e.g. regression coefficient) AND variation (e.g. standard deviation) or associated estimates of uncertainty (e.g. confidence intervals)
☐	☒	For null hypothesis testing, the test statistic (e.g. *F*, *t*, *r*) with confidence intervals, effect sizes, degrees of freedom and *P* value noted<br>*Give P values as exact values whenever suitable.*
☒	☐	For Bayesian analysis, information on the choice of priors and Markov chain Monte Carlo settings
☒	☐	For hierarchical and complex designs, identification of the appropriate level for tests and full reporting of outcomes
☒	☐	Estimates of effect sizes (e.g. Cohen's *d*, Pearson's *r*), indicating how they were calculated

Our web collection on statistics for biologists contains articles on many of the points above.

Software and code

Policy information about availability of computer code

Data collection	Electron microscopy images were captured using CM10 transmission electron microscope. Confocal images were captured using Olympus FV10i-FSW software package (v 4.1) and Zeiss LSM 800 confocal microscope. IHC images were captured using Keyence BZ-X800 microscope using its integrated viewer. Chemiluminescent images were captured using ThermoFisher iBright 1500 imager. GPNMB reporter activity using nano-luciferase was analyzed using Biotek Synergy HT running Gen5 v1.11.5 software. Gene expression by RT-PCR was analyzed using AppliedBiosystems Step One Plus real time PCR system (v2.3). Electron microscopy images were examined with a CM10 transmission electron microscope.
Data analysis	ImageJ (v 1.53a) was used for image analysis. GraphPad Prizm (v 9.5.1) was used for statistical analysis.

For manuscripts utilizing custom algorithms or software that are central to the research but not yet described in published literature, software must be made available to editors and reviewers. We strongly encourage code deposition in a community repository (e.g. GitHub). See the Nature Portfolio guidelines for submitting code & software for further information.

Data

Policy information about availability of data

All manuscripts must include a data availability statement. This statement should provide the following information, where applicable:

- Accession codes, unique identifiers, or web links for publicly available datasets
- A description of any restrictions on data availability
- For clinical datasets or third party data, please ensure that the statement adheres to our policy

All data are available from the corresponding authors upon request and all unique materials generated are readily available from the authors. All RNA sequencing data generated in this study have been deposited to Gene Expression Omnibus (GEO) under accession code GSE244072 and is freely accessible. Source data are provided with this paper in a Source Data file. Uncropped western blots are provided in the Supplementary Information file.

Research involving human participants, their data, or biological material

Policy information about studies with human participants or human data. See also policy information about sex, gender (identity/presentation), and sexual orientation and race, ethnicity and racism.

Reporting on sex and gender	N/A
Reporting on race, ethnicity, or other socially relevant groupings	N/A
Population characteristics	N/A
Recruitment	N/A
Ethics oversight	N/A

Note that full information on the approval of the study protocol must also be provided in the manuscript.

Field-specific reporting

Please select the one below that is the best fit for your research. If you are not sure, read the appropriate sections before making your selection.

☒ Life sciences ☐ Behavioural & social sciences ☐ Ecological, evolutionary & environmental sciences

For a reference copy of the document with all sections, see nature.com/documents/nr-reporting-summary-flat.pdf

Life sciences study design

All studies must disclose on these points even when the disclosure is negative.

Sample size	Sample sizes were selected based on the previous experience and published literature to detect meaningful biological differences.
Data exclusions	No data were excluded from the analyses.
Replication	All experiments were performed with at least in 3 biological replicates and all replication attempts were successful.
Randomization	Randomization was not relevant to this study. All in vitro and in vivo experiments were based on comparisons of samples with defined perturbations (siRNA downregulation, CRISPR-Cas9 inactivation for in vitro experiments) or genotypes (in vivo experiments).
Blinding	The quantification of the IF and IHC experiments was conducted blindly whenever possible based on experimental condition and software-based quantification methods (ImageJ) were used to prevent bias. Investigators were not blinded during experimental group allocations due to a limited number of personnel.

Reporting for specific materials, systems and methods

We require information from authors about some types of materials, experimental systems and methods used in many studies. Here, indicate whether each material, system or method listed is relevant to your study. If you are not sure if a list item applies to your research, read the appropriate section before selecting a response.

Materials & experimental systems

n/a	Involved in the study
☐	☒ Antibodies
☐	☒ Eukaryotic cell lines
☒	☐ Palaeontology and archaeology
☐	☒ Animals and other organisms
☒	☐ Clinical data
☒	☐ Dual use research of concern
☒	☐ Plants

Methods

n/a	Involved in the study
☒	☐ ChIP-seq
☒	☐ Flow cytometry
☒	☐ MRI-based neuroimaging

Antibodies

Antibodies used

The following primary antibodies were used at 1:1000 dilution for immunoblotting and purchased from Cell Signaling Technology unless otherwise indicated:
b-actin (Sigma-Aldrich, A5316)
b-actin (1:5000) (Sigma-Aldrich, A2066)
b-actin (4970)
Vinculin (4650)
GFP (1:5000) (Abcam, ab13970)
Phosphorylated S6 ribosomal protein (pS6 Ser 235/236) (2211)
Total S6 ribosomal protein (TS6) (2217)
TSC2 (4308)
Mouse-specific TFEB (32361)
Human-specific TFEB (37785)
TFE3 (14779)
FLCN (3697)
Phosphorylated TFEB S211 (37681)
RagC (9480)
RagD (Abcam, ab187679)
LAMTOR1 (8975)
Rheb (13879)
Phosphorylated p70 S6 Kinase (pS6K Thr389) (9234) 1:500
p70 S6 Kinase (2708)
4EBP1 (9644)
Phosphorylated 4EBP1 (p4EBP1 Thr37/46) (2855).

The following primary antibodies were used for immunofluorescent detection:

TFEB (1:200) (Cell Signaling cat. 4240),
TFE3 (1:200) (Cell Signaling cat. 14779) and
LAMP1 (H4A3) (1:500) (Santa Cruz cat. 20011).

Secondary antibodies:
ThermoFisher AlexaFluor 568 donkey anti-rabbit, #A10042, 1:500-1:1000
ThermoFisher AlexaFluor 488 donkey anti-rabbit, #A21206, 1:500-1:1000
Amersham/GE Healthcare ECL Mouse IgG, HRP-linked whole Ab (from sheep), #NA931, 1:2000
Amersham/GE Healcare ECL Rabbit IgG, HRP-linked whole Ab (from donkey), NA934, 1:2000
Abcam Goat Anti-Chicken IgY H&L (HRP), #ab97135, 1:5000

Validation

Mouse b-actin (Sigma-Aldrich, A5316), 3759 citations. https://www.sigmaaldrich.com/US/en/product/sigma/a5316. The antibody was validated by the manufacturer for western blotting, immunohistochemistry, indirect ELISA and immunofluorescence. Validation from our study: the antibody produces a band at the expected size on the western blot.

Mouse b-actin (Sigma-Aldrich, A2066), 3135 citations. https://www.sigmaaldrich.com/US/en/search/a2066?focus=products&page=1&perpage=30&sort=relevance&term=a2066&type=product. The antibody was validated by the manufacturer for western blotting, immunohistochemistry and immunofluorescence. Validation from our study: the antibody produces a band at the expected size on the western blot.

Rabbit b-actin (Cell Signaling, 4970S), 5636 citations. https://www.cellsignal.com/products/primary-antibodies/b-actin-13e5-rabbit-mab/4970. The antibody was validated by the manufacturer for western blotting, immunohistochemistry, immunofluorescence and flow cytometry. Validation from our study: the antibody produces a band at the expected size on the western blot.

Vinculin (Cell Signaling, 4650S), 242 citations. https://www.cellsignal.com/products/primary-antibodies/vinculin-antibody/4650. The antibody was validated by the manufacturer for western blotting. Validation from our study: the antibody produces a band at the expected size on the western blot.

GFP (Abcam, ab13970), 3182 citations. https://www.abcam.com/products/primary-antibodies/gfp-antibody-ab13970.html. The antibody was validated by the manufacturer for western blotting and immunofluorescence. Validation from our study: the antibody produces a band at the expected size on the western blot for GFP-fusion proteins.

Phosphorylated S6 Ser 235/236 (Cell Signaling, 2211S), 1446 citations. https://www.cellsignal.com/products/primary-antibodies/phospho-s6-ribosomal-protein-ser235-236-antibody/2211. The antibody was validated by the manufacturer for western blotting, immunohistochemistry, immunofluorescence, flow cytometry and immunoprecipitation. Validation from our study: the antibody produces a band at the expected size on the western blot correlating with mTOR pathway activity.

Total S6 (Cell Signaling, 2217S), 2158 citations. https://www.cellsignal.com/products/primary-antibodies/s6-ribosomal-protein-5g10-rabbit-mab/2217. The antibody was validated by the manufacturer for western blotting, immunohistochemistry and immunofluorescence. Validation from our study: the antibody produces a band at the expected size on the western blot.

Tuberin/TSC2 (Cell Signaling, 4308S), 307 citations. https://www.cellsignal.com/products/primary-antibodies/tuberin-tsc2-d93f12-xp-rabbit-mab/4308. The antibody was validated by the manufacturer for western blotting, immunoprecipitation, immunofluorescence and flow cytometry. Validation from our study: the antibody produces a band at the expected size on the western blot with a decrease in the band intensity after siRNA transfection or complete absence of the band in cells with CRISPR-mediated inactivation.

Mouse-specific TFEB (Cell Signaling, 32361S), 17 citations. https://www.cellsignal.com/products/primary-antibodies/tfeb-d4l2p-rabbit-mab/32361. The antibody was validated by the manufacturer for western blotting and immunoprecipitation. Validation from our study: the antibody produces a band at the expected size on the western blot.

Human-specific TFEB (Cell Signaling, 37785S), 44 citations. https://www.cellsignal.com/products/primary-antibodies/tfeb-d2o7d-rabbit-mab/37785. The antibody was validated by the manufacturer for western blotting, immunohistochemistry, immunoprecipitation and chromatin immunoprecipitation. Validation from our study: the antibody produces a band at the expected size on the western blot.
TFE3 (Cell Signaling, 14779S), 41 citations (currently discontinued). https://www.cellsignal.com/product/productDetail.jsp?productId=14779. The antibody was validated by the manufacturer for western blotting. Validation from our study: the antibody produces a band at the expected size on the western blot.

FLCN (Cell Signaling, 3697S), 49 citations. https://www.cellsignal.com/products/primary-antibodies/flcn-d14g9-rabbit-mab/3697. The antibody was validated by the manufacturer for western blotting and immunoprecipitation. Validation from our study: the antibody produces a band at the expected size on the western blot.

Phosphorylated TFEB Ser211 (Cell Signaling, 37681S), 31 citations. https://www.cellsignal.com/products/primary-antibodies/phospho-tfeb-ser211-e9s8n-rabbit-mab/37681. The antibody was validated by the manufacturer for western blotting. Validation from our study: the antibody produces a band at the expected size on the western blot and band intensity correlating with subcellular localization of TFEB.
RagC (Cell Signaling, 9480S), 25 citations. https://www.cellsignal.com/products/primary-antibodies/ragc-d8h5-rabbit-mab/9480. The antibody was validated by the manufacturer for western blotting, immunofluorescence, immunoprecipitation and flow cytometry. Validation from our study: the antibody produces a band at the expected size on the western blot.
RagD (Abcam, ab187679), 3 citations. https://www.abcam.com/products/primary-antibodies/rag-d-antibody-n-terminal-ab187679.html. The antibody was validated by the manufacturer for western blotting and immunoprecipitation. Validation from our study: the antibody produces a band at the expected size on the western blot.

LAMTOR1 (Cell Signaling, 8975S), 65 citations. https://www.cellsignal.com/products/primary-antibodies/lamtor1-c11orf59-d11h6-xp-rabbit-mab/8975. The antibody was validated by the manufacturer for western blotting, immunoprecipitation, immunohistochemistry and immunofluorescence. Validation from our study: the antibody produces a band at the expected size on the western blot.

Rheb (Cell Signaling, 13879S), 54 citations. https://www.cellsignal.com/products/primary-antibodies/rheb-e1g1r-rabbit-mab/13879. The antibody was validated by the manufacturer for western blotting and immunoprecipitation. Validation from our study: the antibody produces a band at the expected size on the western blot with a decrease in the band intensity after siRNA transfection.

Phosphorylated p70 S6 Kinase Thr389 (Cell Signaling, 9234S), 1754 citations. https://www.cellsignal.com/products/primary-antibodies/phospho-p70-s6-kinase-thr389-108d2-rabbit-mab/9234. The antibody was validated by the manufacturer for western blotting. Validation from our study: the antibody produces a band at the expected size on the western blot correlating with mTOR pathway activity.

p70 S6 Kinase (Cell Signaling, 2708S), 1551 citations. https://www.cellsignal.com/products/primary-antibodies/p70-s6-kinase-49d7-rabbit-mab/2708. The antibody was validated by the manufacturer for western blotting. Validation from our study: the antibody produces a band at the expected size on the western blot.

4EBP1 (Cell Signaling, 9644S), 1166 citations. https://www.cellsignal.com/products/primary-antibodies/4e-bp1-53h11-rabbit-mab/9644. The antibody was validated by the manufacturer for western blotting, immunoprecipitation, immunohistochemistry, immunofluorescence and flow cytometry. Validation from our study: the antibody produces a band at the expected size on the western blot.

Phosphorylated 4EBP1 (p4EBP1 Thr37/46) (Cell Signaling, 2855S), 1684 citations. https://www.cellsignal.com/products/primary-antibodies/phospho-4e-bp1-thr37-46-236b4-rabbit-mab/2855. The antibody was validated by the manufacturer for western blotting, immunohistochemistry, immunofluorescence and flow cytometry. Validation from our study: the antibody produces a band at the

expected size on the western blot correlating with mTOR pathway activity.

TFEB (Cell Signaling, 4240). 166 citations. https://www.cellsignal.com/products/primary-antibodies/tfeb-antibody/4240. The antibody was validated by the manufacturer for western blotting and immunoprecipitation. Validation from our study: the antibody produces a band at the expected size on the western blot.

LAMP1 (Santa Cruz, 20011), 460 citations. https://www.scbt.com/p/lamp-1-antibody-h4a3. The antibody was validated by the manufacturer for western blotting, immunoprecipitation, immunohistochemistry, immunofluorescence and flow cytometry. Validation from our study: the antibody produces a band at the expected size on the western blot and punctate pattern on IF consistent with lysosomal localization.

Eukaryotic cell lines

Policy information about cell lines and Sex and Gender in Research

Cell line source(s)	HeLa cells and HEK293T cells were purchased from ATCC. Hela TFEB-GFP cells were developed by the Shawn Ferguson lab (PMID: 22692423). 621-102 and 621-103 cells were developed and characterized previously by the Henske Lab (PMID: 12922981).
Authentication	Knockout and knockdown cells were authenticated by WB or RT-PCR. Overexpression cells were authenticated by WB or RT-PCR. Cell lines purchased from ATCC were kept in culture using all the standard methods to avoid contamination with other cell lines and validated by morphological analysis.
Mycoplasma contamination	Cells were tested for mycoplasma on a regular basis (every 2-3 months) and were confirmed negative.
Commonly misidentified lines (See ICLAC register)	No commonly misidentified cells were used in this study.

Animals and other research organisms

Policy information about studies involving animals; ARRIVE guidelines recommended for reporting animal research, and Sex and Gender in Research

Laboratory animals	All mice in the study were in C57BL6 background and housed in an animal facility with 12h light/12h dark cycle at 72°F and 40% humidity with ad libitum access to food and water unless otherwise specified in the appropriate figure legends. Mice were maintained on irradiated PicoLab Rodent Diet 20 (5053). The Tfeb fl/fl mice were developed by Andrea Ballabio. Tsc2 fl/fl, CaggCreERT2, and KspCre (Cadh16Cre) mice were acquired from Jackson Laboratories. 50 day old KspCre animals of the indicated genotypes were used for electron microscopy of the kidneys.
Wild animals	No wild animals were used.
Reporting on sex	Experimental data (such as survival etc.) were analyzed based on gender and reported in the manuscript when statistically significant
Field-collected samples	No field-collected samples were used.
Ethics oversight	Animal studies were approved by the Brigham and Women's Hospital Animal Care and Use Committee.

Note that full information on the approval of the study protocol must also be provided in the manuscript.
