# Supplementary material for: TFEB drives mTORC1 hyperactivation and kidney disease in Tuberous Sclerosis Complex
Source: Nat Commun. 2024 Jan 9;15:406. doi: 10.1038/s41467-023-44229-4 (PMC10776564; doi:10.1038/s41467-023-44229-4)
Supplement: Supplementary file 2 — Peer Review File [file 41467_2023_44229_MOESM2_ESM.pdf]

nature portfolio

Peer Review File

TFEB drives mTORC1 hyperactivation and kidney disease in Tuberous Sclerosis Complex

Open Access This file is licensed under a Creative Commons Attribution 4.0 International License, which permits use, sharing, adaptation, distribution and reproduction in any medium or format, as long as you give appropriate credit to the original author(s) and the source, provide a link to the Creative Commons license, and indicate if changes were made. In the cases where the authors are anonymous, such as is the case for the reports of anonymous peer reviewers, author attribution should be to 'Anonymous Referee' followed by a clear attribution to the source work. The images or other third party material in this file are included in the article's Creative Commons license, unless indicated otherwise in a credit line to the material. If material is not included in the article's Creative Commons license and your intended use is not permitted by statutory regulation or exceeds the permitted use, you will need to obtain permission directly from the copyright holder. To view a copy of this license, visit http://creativecommons.org/licenses/by/4.0/.

REVIEWER COMMENTS

Reviewer #1 (Remarks to the Author):

In this study the authors showed that TFEB drives mTORC1 hyperactivation in kidney disease occuring in tuberous sclerosis complex suggesting the potential role of TFEB inhibitors in this particular setting.

Comments:

Regarding the role of GPNMB, the authors claimed its value as histological marker of TSC2. However, GPNMB has been found in other tumors not related to TSC2. Moreover,I suggest to add cathepsin k as an important protein in this scenario as previously suggested (PMID 21874011, PMID 34069976)

The authors should include and discuss their results based on the recent manuscript (PMID 36697823)

Reviewer #2 (Remarks to the Author):

TFEB drives mTORC1 hyperactivation and kidney disease in Tuberous Sclerosis Complex

My decision: Acceptable

My comments:

TSC is caused by germline TSC1 or TSC2 mutations. Hyperactivation of mTORC1 plays a role in tumor formation.

The authors showed that TFEB is the primary driver of kidney tumorigenesis in two mouse models of TSC.

They indicated that mTORC1 hyperactivation in TSC requires activation of TFEB. They concluded that TFEB inhibition as a potential therapeutic strategy for TSC.

The paper has new findings and useful.

Reviewer #3 (Remarks to the Author):

This study builds on recent prior work from several groups showing that TFEB is unexpectedly hypo-phosphorylated and activated with TSC2 loss, despite mTOR activation. Using two transgenic mouse models, the authors demonstrate that TFEB knockout is sufficient to reverse the enlarged renal phenotype and altered gene expression associated with perinatal complete TSC2 loss in the kidney epithelium and may also be associated with decreased mTORC1 activation. If the latter finding is true, this would suggest that TFEB activation may be necessary for mTORC1 activity in the context of TSC2 loss, a rather provocative finding. Though a role for TFEB in mTORC1 activation has been previously described by this group (PMID 28619945), this has not been extended to the specific context of TSC2 loss. While these findings are interesting and clinically relevant, significant additional work will be necessary to elucidate the mechanism by which TFEB, a transcription factor, may regulate mTORC1 activation downstream of TSC2 loss. In addition, there are a number of issues around experimental rigor that need to be addressed throughout.

Specific comments:

1) Figure 5 contains one of the more provocative findings in the study—that mTORC1 activity diminished with TFEB loss in TSC2 KO kidneys in vivo. However, some additional work is necessary to substantiate and further probe this finding. p-4EBP1 and total 4EBP1 are concomitantly increased with TSC2 loss and both decreased with additional TFEB knockout. Thus, the phospho-to-total ratio does not change across these tissues, which raises questions about whether mTORC1 activity is truly changing.

a. Additional blots for p-P70S6K and p-S6 (and totals) should be added to Figure 5 to show the effects on these substrates. Immunoblotting for TSC2 and TFEB must also be added to this figure. P-S6 immunohistochemistry to replicate the finding in situ would also be important.

b. Quantification of all immunoblots across triplicate samples is necessary for rigor.

c. Addition of samples from the CaggCreERT2 model is important to replicate these results in another system.

d. The finding looks much less impressive in HeLa cells—quantification of these blots is critical as is replication in the HEK293 model or another cell line model. Addition of p-4EBP1 and total is also necessary.

e. Gene expression profiling in both mouse models should be assessed to verify that mTOR signaling-related gene sets show significant negative enrichment in the DKO model compared to the TSC2 KO model. This will be critical orthogonal validation of the WB findings. Is the same negative enrichment seen in the TFEB KO model compared to CTRL?

2) If the finding truly holds up after completing experiments in #1, what is the mechanism for the decrease in mTORC1 activity with TFEB loss in the context of TSC2? Is this due to RagD downregulation as previously shown by this group? Or is there another mechanism? This is critical to flesh out significantly to add novelty to the manuscript.

3) What is the role for TFE3? The findings in this study would seem to suggest that TFE3 is not functionally redundant with TFEB for the kidney phenotype or mTORC1 activation with TSC2 loss which is somewhat surprising given redundancy seen in multiple other contexts. Is TFE3 activated with TSC2 loss in the kidney?

4) The murine models used for TSC2 are not clinically relevant since both lead to bi-allelic TSC2 loss in the majority of kidney cells from an early age. This does not model the typical stochastic second hit mechanism seen in tuberous sclerosis-associated renal tumors, and the kidney phenotype (enlarged, cystic) is not typical of tuberous sclerosis. At a minimum, this should be acknowledged in the Discussion.

a. It would be more convincing to examine TFEB inactivation in the A/J mouse model which develops spontaneous renal tumors with age similar to humans with tuberous sclerosis.

b. What is the cause of the kidney enlargement in the CaggCreERT2 model? The kidneys do not look cystic from the images provided. Is there any increase in cell mass overall? Proliferation? It would also be important to add in survival curves for this model with and without Tfeb KO.

5) For appropriate rigor, all IF experiments in the manuscript must be accompanied by quantifications across three biological replicates. Murine experiments should be separately reported for male and female sexes in order to account for sex as a biological variable.

6) Figure 1 is replicative of Figure 2 and can also be moved to Extended figures. Figure 3 is largely replicative of reference 27 and should be moved to Extended figures.

7) Figure 1e and 1f, 2f and 2g as well as 4d and 4e require statistical analysis. Changes in heat map with DKO are not very impressive visually.

Reviewer #4 (Remarks to the Author):

The manuscript by Alesi et al. investigates the activity and functional role of the TFEB transcription factor in kidney models of Tuberous Sclerosis Complex (TSC), as well as in TSC mutant cell lines. Two important and convincing conclusions are supported by the experimental evidence: 1) TFEB deletion rescues the kidney cysts and failure in TSC mouse models; 2) TFEB is hypophosphorylated and activated in TSC cells, with rapamycin being effective in counteracting this activation.

The manuscript is rather succinct. Although many experiments (as always) could be added to better characterize the model, the above-mentioned conclusions stand out for their relevance and are well documented. The following data would strengthen the study:

1) Torin and Rapamycin could be used to dissect the contribution of canonical vs. non-canonical mTOR substrates in the kidney phenotype. Both drugs inhibit canonical substrates such as S6K. In contrast, Rapamycin moves TFEB out of the nucleus while Torin potentiates TFEB action. Read-outs of kidney cysts and TFEB gene expression could be compared after Rapamycin and Torin treatment to determine whether TFEB acts by potentiating mTOR signaling through canonical substrates or whether has some direct activities promoting cysts. The second possibility, but not the first, would justify the conclusion that "in the murine kidney, the non-canonical substrate TFEB predominates".

2) Along this line, the Figure 5 requires further analysis. The interpretation that TFEB deletion impairs mTORC1 up-regulation of 4EBP phosphorylation is confounded by the concomitant effect on 4EBP total protein levels. In Figure 5B, total 4EBP levels decrease in TFEB mutants, with no difference in the electrophoretic mobility, which is surprising if the phosphorylation was profoundly affected. P-S6K and P-S6 should also be analyzed, together with p-Akt (mTORC2 read-out) and total TFEB levels. Analysis of kidneys from younger mice may be more straightforward, as the cell composition is less affected.

3) In the text, the authors should discuss some hypothesis explaining why rapamycin treatment may increase TFEB phosphorylation and nuclear exclusion in TSC cells.

Point-by-point response to reviewers' comments (NCOMMS-23-03365-T).

Reviewer #1 (Remarks to the Author):

In this study the authors showed that TFEB drives mTORC1 hyperactivation in kidney disease occurring in tuberous sclerosis complex suggesting the potential role of TFEB inhibitors in this particular setting.
Comments:
1. Regarding the role of GPNMB, the authors claimed its value as histological marker of TSC2. However, GPNMB has been found in other tumors not related to TSC2. Moreover, I suggest to add cathepsin k as an important protein in this scenario as previously suggested (PMID 21874011, PMID 34069976).

Response: We agree that GPNMB expression cannot be used as a sole histological marker of TSC-associated tumors. In our work, GPNMB is used as an indicator of activation of TFE3/TFEB. Our RNA sequencing data from KspCre; Tsc2fl/fl mice shows that Cathepsin K is elevated by ~50% (see Figure below), consistent with prior work showing that Cathepsin K is a marker of TSC-associated tumors. We have included the suggested references to the discussion to clarify the role of Cathepsin K as marker of TSC: "Indeed, MITF is considered a hallmark gene of lymphangioleiomyomatosis (LAM) and angiomyolipomas in TSC, based on upregulation of genes that are known transcriptional targets of MITF/TFE3/TFEB including Cathepsin K."

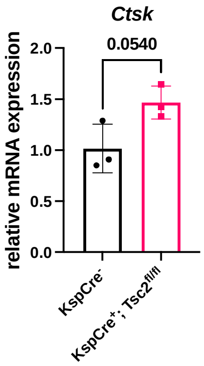

2. The authors should include and discuss their results based on the recent manuscript (PMID 36697823).

Response: This paper revealing the cryo-EM structure of the lysosomal-mTORC1-Rag-Ragulator complex, was published while our manuscript was under review. We agree that it is important, and updated the discussion with the following paragraph:
"Our data presented here support and contribute to the understanding of a distinction between the canonical substrates of mTORC1 (including S6K and 4EBP1) and the non-canonical substrates (including TFEB and TFE3). In TSC, the canonical substrates are hyperphosphorylated and the non-canonical substrates are, surprisingly, hypophosphorylated, leading to increased nuclear TFEB/TFE3. Recently, a cryo electron microscopy structure of the lysosomal TFEB-mTORC1-Rag-Ragulator megacomplex was published, revealing the structural basis of the differentiation between canonical and non-canonical mTOR substrates."

Reviewer #2 (Remarks to the Author):

TFEB drives mTORC1 hyperactivation and kidney disease in Tuberous Sclerosis Complex

My decision: Acceptable
My comments:

TSC is caused by germline TSC1 or TSC2 mutations. Hyperactivation of mTORC1 plays a role in tumor formation.
The authors showed that TFEB is the primary driver of kidney tumorigenesis in two mouse models of TSC.
They indicated that mTORC1 hyperactivation in TSC requires activation of TFEB. They concluded that TFEB inhibition as a potential therapeutic strategy for TSC.

The paper has new findings and useful.

Response: We thank the Reviewer for the positive feedback.

Reviewer #3 (Remarks to the Author):

This study builds on recent prior work from several groups showing that TFEB is unexpectedly hypo-phosphorylated and activated with TSC2 loss, despite mTOR activation. Using two transgenic mouse models, the authors demonstrate that TFEB knockout is sufficient to reverse the enlarged renal phenotype and altered gene expression associated with perinatal complete TSC2 loss in the kidney epithelium and may be associated with decreased mTORC1 activation. If the latter finding is true, this would suggest that TFEB activation may be necessary for mTORC1 activity in the context of TSC2 loss, a rather provocative finding. Though a role for TFEB in mTORC1 activation has been previously described by this group (PMID 28619945), this has not been extended to the specific context of TSC2 loss. While these findings are interesting and clinically relevant, significant additional work will be necessary to elucidate the mechanism by which TFEB, a transcription factor, may regulate mTORC1 activation downstream of TSC2 loss. In addition, there are a number of issues around experimental rigor that need to be addressed throughout.

Specific comments:

1) Figure 5 [now Figure 6] contains one of the more provocative findings in the study—that mTORC1 activity diminished with TFEB loss in TSC2 KO kidneys in vivo. However, some additional work is necessary to substantiate and further probe this finding. p-4EBP1 and total 4EBP1 are concomitantly increased with TSC2 loss and both decreased with additional TFEB knockout. Thus, the phospho-to-total ratio does not change across these tissues, which raises questions about whether mTORC1 activity is truly changing.

Response: To further understand mTORC1 activity we looked at phospho-S6K (as suggested). At P50 and P90 phospho-S6K is increased in Tsc2 KO kidneys and decreased in the DKO kidneys. In addition, total levels of proteins critical for mTOR activity (RagC, RagD, and LAMTOR1, Extended Data Fig. 7b and c) are upregulated in the Tsc2 KO kidneys and decreased in the double KO kidneys.
In an important new experiment suggested by Reviewer 4, we analyzed the kidneys of the Ksp-Cre mice at p15, prior to the onset of substantial kidney pathology. At this early timepoint, levels of total 4EBP1 and S6K are only slightly increased in the Tsc2 KO, while phospho-4EBP1 and phospho-S6K are markedly increased in the Tsc2 KO and normalized in the Tsc2/TFEB DKO (Figure 6a).
We next examined another indicator of mTORC1 activity, Mthfd2 (methylenetetrahydrofolate dehydrogenase 2) expression. Mthdf2 was shown by Brendan Manning's group to be strongly associated with mTORC1 activity (PMID 26912861). Mthfd2 was increased ~5 fold in the Ksp-Cre kidneys and the CAGG-Cre Tsc2 KO kidneys (Fig.6d) and decreased by more than 50% in the Tsc2/Tfeb double KO, consistent with TFEB-dependent mTORC1 activity.
We agree that it is interesting that total 4EBP1 is increased in the Ksp-Cre Tsc2 KO kidneys at P50 and P90 (Figure 6b and c). Most importantly, both total and phospho-4EBP1 are decreased in the Tsc2/Tfeb DKO kidneys at P50 and P90. In new data, we analyzed the CaggCreERT2 model and, similarly to the Ksp model, found that both total and phospho-4EBP1 are increased in the Tsc2 KO kidneys at P30 and normalized by Tfeb knockdown (Extended Data Fig. 7d).
Taken together, these data further support a model in which TFEB drives mTORC1 activation in TSC.

a. Additional blots for p-P70S6K and p-S6 (and totals) should be added to Figure 5 to show the effects on these substrates. Immunoblotting for TSC2 and TFEB must also be added to this figure. P-S6 immunohistochemistry to replicate the finding in situ would also be important.

Response: As suggested, we tested total and phospho-S6K, which are both elevated at P15, P50 and P90, and normalized in the double knockout. We were unable to obtain consistent results with phospho-S6 by either immunoblotting or IHC.

b. Quantification of all immunoblots across triplicate samples is necessary for rigor.

Response: We performed quantification across triplicate samples and added these to Figs. 3c, 3g, 4b, 4d, 6e, 6f, Extended Data Figs. 5a, 5b, 6b, 6c, 7b, 7c. Fig. 6a is quantified in Extended Data Fig 7a.

c. Addition of samples from the CaggCreERT2 model is important to replicate these results in another system.

Response: In the new Extended Figure 7d, we show that phospho-4EBP1 and total 4-EBP1 are increased in the CaggCreERT2 model, similarly to the Ksp-Cre model.

d. The finding looks much less impressive in HeLa cells—quantification of these blots is critical as is replication in the HEK293 model or another cell line model. Addition of p-4EBP1 and total is also necessary.

Response: We agree that the findings in the mice are much more impressive than the results in HeLa cells.
We added quantification to the HeLa results, which shows that the ratio of phospho-S6K to total S6K is decreased by 50% ($p<0.01$) in the Tsc2-KO cells treated with TFEB siRNA (Fig. 6e). We added a second cell line model, 621-102 cells, which were derived from a TSC2-deficient human angiomyolipoma. Downregulation of TFEB, but not TFE3, decreased the ratio of phospho-S6K to total S6K by 70% ($p<0.05$, new Fig. 6f), similarly to the HeLa results.
The reasons for the differences between the in vivo and in vitro results are unclear to us. In vivo factors such as the immune microenvironment or oxygen/nutrient availability may be involved.

e. Gene expression profiling in both mouse models should be assessed to verify that mTOR signaling-related gene sets show significant negative enrichment in the DKO model compared to the TSC2 KO model. This will be critical orthogonal validation of the WB findings. Is the same negative enrichment seen in the TFEB KO model compared to CTRL?

Response: As suggested, we analyzed mTOR signaling related genes in the RNA-sequencing data set, revealing that multiple components of mTOR signaling including RagC, RagD, and LAMTOR1, are increased in the Tsc2 KO kidneys and decreased in the DKO kidneys. We confirmed that the proteins are upregulated in the Tsc2 KO kidneys and decreased in the Tsc2/Tfeb DKO kidneys (Fig. 6a, Extended data Fig. 7a-c).
As noted above, we also analyzed the expression of Mthfd2, which was established as an indicator of mTORC1 activity by Brendan Manning's group (PMID 26912861). Mthfd2 is increased ~5 fold in both the Ksp-Cre and CAGGCre Tsc2 KO kidneys (Fig.6d) and decreased by more than 50% in the Tsc2/Tfeb double KO, consistent with TFEB-dependent mTORC1 activity.
The same negative enrichment is not seen in Tfeb KO vs CTRL mice, which was expected since in order to be hyperactive TFEB needs to be nuclear, a condition we mainly observed in TSC2-deficiency.

2) If the finding truly holds up after completing experiments in #1, what is the mechanism for the decrease in mTORC1 activity with TFEB loss in the context of TSC2? Is this due to RagD downregulation as previously shown by this group? Or is there another mechanism? This is critical to flesh out significantly to add novelty to the manuscript.

Response: Tsc2 loss in the Ksp-Cre model leads to increased protein levels of RagC, RagD and LAMTOR1 (Fig. 6a, Extended data Fig. 7a-c), with RagD protein level increased 4-fold at P50 and 10-fold at P90 (Extended Data Fig. 7b, c). In the Tsc2/Tfeb DKO, levels of all three proteins are decreased (at P90, RagD is decreased by ~80%). Based on these data, we hypothesize that at least one mechanism through which TFEB regulates mTORC1 activity in TSC is through TFEB-dependent upregulation of RagC and RagD.

3) What is the role for TFE3? The findings in this study would seem to suggest that TFE3 is not functionally redundant with TFEB for the kidney phenotype or mTORC1 activation with TSC2 loss which is somewhat surprising given redundancy seen in multiple other contexts. Is TFE3 activated with TSC2 loss in the kidney?

Response: We agree that the question of redundancy between TFEB and TFE3 is important. The striking effects of genetic loss of TFEB in our models suggest that TFEB may play a dominant role in the kidney compared to the other members of the MiTF family. Interestingly, as noted above, in human TSC2-deficient renal angiomyolipoma-derived cells, downregulation of TFEB but not TFE3 decreases phospho-S6K (Fig. 6f). Multiple groups have shown that TFE3 is nuclear and active in models of TSC. Whether TFE3 impacts the kidney phenotypes in these models is unknown. Interestingly, in a Ksp-driven model in which FLCN was deleted, levels of TFEB are higher than TFE3, suggesting that the relative levels are important (PMID:36987696).

4) The murine models used for TSC2 are not clinically relevant since both lead to bi-allelic TSC2 loss in the majority of kidney cells from an early age. This does not model the typical stochastic second hit mechanism seen in tuberous sclerosis-associated renal tumors, and the kidney phenotype (enlarged, cystic) is not typical of tuberous sclerosis. At a minimum, this should be acknowledged in the Discussion.

Response: We agree that these mouse models do not replicate the typical stochastic two-hit mechanism seen in TSC. Regarding cysts, many TSC patients have renal cysts, but they are not usually as numerous as in these mouse models, with the exception of patients with contiguous deletion of TSC2 and PKD1 (adjacent genes on chromosome 16p13), who can develop severe renal cystic disease in early childhood.
As suggested, we added the following to the Discussion:
"The mouse models of Tsc2 inactivation presented here do not replicate the typical genetic mechanisms of kidney disease in human TSC, in which a germline "first hit" mutation is followed by a stochastic "second hit" mutation, but they clearly indicate that in the mouse kidney, TFEB plays a critical role in TSC-associated pathology."

a. It would be more convincing to examine TFEB inactivation in the A/J mouse model which develops spontaneous renal tumors with age similar to humans with tuberous sclerosis.

Response: We agree that TFEB inactivation in the Tsc2+/- mice (in the A/J genetic background) would be of interest. These mice have a delayed phenotype (with tumors best assessed at 12 mo of age) and in our hands are currently breeding poorly, making this experiment quite challenging.

b. What is the cause of the kidney enlargement in the CaggCreERT2 model? The kidneys do not look cystic from the images provided. Is there any increase in cell mass overall? Proliferation? It would also be important to add in survival curves for this model with and without Tfeb KO.

Response: The kidneys in the CAGGCreERT2 model are cystic at P30. We have added new H&E images to better illustrate this (Figure 1c, new panels). The kidneys-to-body weight ratios were increased (Figure 1d, consistent with increased cell mass). As suggested, we stained for Ki67 to assess proliferation and found no significant differences between genotypes at P30. We added the survival curve for this model as well (Extended data Fig. 1d).

5) For appropriate rigor, all IF experiments in the manuscript must be accompanied by quantifications across three biological replicates. Murine experiments should be separately reported for male and female sexes in order to account for sex as a biological variable.

Response: We added quantification for all IF experiments.
As suggested, we analyzed all mouse experiments for males and females separately. No significant differences were seen in kidneys/body weight ratio in the CAGGCre model (P30) or KSP-Cre model (P50). Interestingly, in the KSP-Cre model, males have poorer survival, with only 35% of males surviving to P90 vs. 75% of females. These data were added to the new Fig. 2e.

6) Figure 1 is replicative of Figure 2 and can also be moved to extended figures. Figure 3 is largely replicative of reference 27 and should be moved to Extended figures.

Response: Regarding Fig. 1 and Fig. 2, we feel that both in vivo models are important, since the CAGGCre model is a whole body model, and loss of TSC2 is induced in the early post-natal period (P1-3), while the KSP-Cre model has constitutive TSC2 loss and is kidney-specific. Both of these models are novel in the TSC field. Regarding Fig. 3, as suggested we moved our HEK293T data to Extended Figures (Extended Data Fig 5), but we think our Rheb knockdown data instead are entirely novel.

7) Figure 1e and 1f, 2f and 2g as well as 4d and 4e require statistical analysis. Changes in heat map with DKO are not very impressive visually.

Response: We added a supplemental Table with the statistical analysis of each lysosomal gene shown in Figures 1e, 2g, and 5e. The Venn diagrams in Figures 1f, 2f, and 5d include only genes that are significantly different ($p<0.05$) between the indicated genotypes using a T test.

Reviewer #4 (Remarks to the Author):

The manuscript by Alesi et al. investigates the activity and functional role of the TFEB transcription factor in kidney models of Tuberous Sclerosis Complex (TSC), as well as in TSC mutant cell lines. Two important and convincing conclusions are supported by the experimental evidence: 1) TFEB deletion rescues the kidney cysts and failure in TSC mouse models; 2) TFEB is hypophosphorylated and activated in TSC cells, with rapamycin being effective in counteracting this activation.
The manuscript is rather succinct. Although many experiments (as always) could be added to better characterize the model, the above-mentioned conclusions stand out for their relevance and are well documented. The following data would strengthen the study:
1) Torin and Rapamycin could be used to dissect the contribution of canonical vs. non-canonical mTOR substrates in the kidney phenotype. Both drugs inhibit canonical substrates such as S6K. In contrast, Rapamycin moves TFEB out of the nucleus while Torin potentiates TFEB action. Read-outs of kidney cysts and TFEB gene expression could be compared after Rapamycin and Torin treatment to determine whether TFEB acts by potentiating mTOR signaling through canonical substrates or whether has some direct activities promoting cysts. The second possibility, but not the first, would justify the conclusion that "in the murine kidney, the non-canonical substrate TFEB predominates".

Response: We agree that evaluating the differential effects of Rapamycin and Torin1 in vivo could provide additional evidence to dissect the contributions of the canonical vs. non-canonical mTOR substrates in the kidney. We thank the reviewer for the excellent suggestion and we hope to include these experiments in future work considering that they will take longer time, since, we would need to begin with dose-finding studies to assess efficacy (in terms of targets inhibition) and toxicity (Torin1 is more toxic than Rapamycin), prior to comparisons of Rapamycin and Torin1 in terms of renal pathology.

2) Along this line, the Figure 5 requires further analysis. The interpretation that TFEB deletion impairs mTORC1 up-regulation of 4EBP phosphorylation is confounded by the concomitant effect on 4EBP total protein levels. In Figure 5B, total 4EBP levels decrease in TFEB mutants, with no difference in the electrophoretic mobility, which is surprising if the phosphorylation was profoundly affected.

Response: We agree that the concomitant increase in phospho-4EBP1 and total 4EBP1 confounds these results. We have already answered the same question by Reviewer number 3 (please see above). Regarding the electrophoretic mobility of total 4EBP1, this is difficult for us to quantitate because the signal is faint in the double knockout kidneys. A mobility trend is present in the phospho-4EBP1 blots.

P-S6K and P-S6 should also be analyzed, together with p-Akt (mTORC2 read-out) and total TFEB levels.

Response: As suggested, we tested total and phospho-S6K, which are both elevated at P15, P50 and P90, and normalized in the double knockout. We were unable to obtain consistent results with phospho-S6 by either immunoblotting or IHC.
Regarding Akt, as suggested we measured phospho-Akt (S473) in the Ksp-Cre kidney lysates at P90, but the results were quite variable with no clear trends.
To further examine whether TFEB regulates mTORC1 activity in TSC, we analyzed expression of Mthfd2 (methylenetetrahydrofolate dehydrogenase 2). Mthfd2 was shown by Brendan Manning's group to be strongly associated with mTORC1 activity (PMID 26912861). Mthfd2 was increased 4.5-5-fold in the Ksp-Cre CAGG-Cre kidneys (Fig.6d) and decreased by more than 50% in the double KO, consistent with TFEB-dependent mTORC1 activity.
Regarding total TFEB levels, by RT-PCR in the Ksp-Cre kidneys at P90, TFEB is knocked down about 50% and TSC2 is decreased about 75% (new Extended Data Fig. 2a). For the CaggCreERT2 mice at p30, TFEB is knocked down about 70% and TSC2 about 80% (Extended Data Fig. 1b).

Analysis of kidneys from younger mice may be more straightforward, as the cell composition is less affected.

Response: As suggested, we harvested a new cohort of mice at P15, and found that phospho-4EBP1 and phospho-S6K are markedly increased in the Tsc2 KO kidneys and decreased in the Tsc2/Tfeb DKO, while total 4EBP1 and total S6K are only slightly elevated in the Tsc2 KO kidneys (Fig. 6a). We thank the Reviewer suggesting this experiment, which further supports a TFEB-dependent increase in mTORC1 activity in TSC.

3) In the text, the authors should discuss some hypotheses explaining why rapamycin treatment may increase TFEB phosphorylation and nuclear exclusion in TSC cells.

Response: We have included the following in the discussion:
"The mechanisms underlying the unexpected Rapamycin and Rheb downregulation-induced nuclear exclusion and increase in TFEB phosphorylation remain unclear. We hypothesize that in TSC, mTORC1 inhibition may impair the FLCN-RagC axis and/or lead to a conformational change in mTORC1, resulting in increased phosphorylation and nuclear exclusion of TFEB. "

In closing, we thank the Reviewers for their positive feedback and suggestions which have helped us strengthen the manuscript. We believe that these findings will have a substantial impact on the TSC field and on our understanding of the complex relationship between mTORC1 and TFEB.

REVIEWERS' COMMENTS

Reviewer #1 (Remarks to the Author):

The authors fully adressed my comments

The paper i now acceptable for me

Reviewer #3 (Remarks to the Author):

The authors have done a very nice job of addressing my comments, the immunoblot from the P15 experiment is much more convincing that mTOR activity is decreased with TFEB knockout.

Reviewer #4 (Remarks to the Author):

The authors addressed most of the previously raised issues and the manuscript should be accepted for publication. Since the comparison between rapamycin and torin could not be addressed to dissect canonical vs non-canonical substrates (point #1 in my previous report), the authors should state in the discussion that this future experiment will be needed before concluding that “in the murine kidney, the non-canonical substrate TFEB predominates”.

Below, we address the specific comment of the reviewer 4.

Reviewer #4

1. *The authors addressed most of the previously raised issues and the manuscript should be accepted for publication. Since the comparison between rapamycin and torin could not be addressed to dissect canonical vs non-canonical substrates (point #1 in my previous report), the authors should state in the discussion that this future experiment will be needed before concluding that "in the murine kidney, the non-canonical substrate TFEB predominates".*

Response: We thank the Reviewer for the positive feedback. We now added a following paragraph in the Discussion:

Future experiments comparing Rapamycin and Torin1 treatment could help to further dissect the canonical versus non-canonical substrate predominance in our mouse models of TSC. In TSC-deficient cells, we have shown that Rapamycin inhibits both canonical and non-canonical substrates, by moving TFEB/TFE3 out of the nucleus. In contrast, Torin inhibits only the canonical substrates, since TFEB remains nuclear upon Torin treatment.
